# FGF21 alleviates pulmonary hypertension by inhibiting mTORC1/EIF4EBP1 pathway via H19

**DOI:** 10.1111/jcmm.17318

**Published:** 2022-04-19

**Authors:** Xiuchun Li, Yaxin Zhang, Lihuang Su, Luqiong Cai, Chi Zhang, Jianhao Zhang, Junwei Sun, Mengyu Chai, Mengsi Cai, Qian Wu, Chi Zhang, Xiaoqing Yan, Liangxing Wang, Xiaoying Huang

**Affiliations:** ^1^ Division of Pulmonary Medicine the First Affiliated Hospital of Wenzhou Medical University Key Laboratory of Heart and Lung Wenzhou P.R. China; ^2^ 26453 The First Clinical Medical College Wenzhou Medical University Wenzhou P.R. China; ^3^ 26453 Chinese‐American Research Institute for Diabetic Complications at Department of Pharmacy Wenzhou Medical University Wenzhou P.R. China

**Keywords:** fibroblast growth factor 21, long non‐coding RNAs, molecular mechanism, pulmonary hypertension, pulmonary vascular diseases

## Abstract

Long non‐coding RNAs (lncRNAs) play a significant role in pulmonary hypertension (PH). Our preliminary data showed that hypoxia‐induced PH is attenuated by fibroblast growth factor 21 (FGF21) administration. Therefore, we further investigated the regulatory role of long non‐coding RNAs in PH treated with FGF21. RNA sequencing analysis and real‐time PCR identified a significantly up‐regulation of the H19 after FGF21 administration. Moreover, gain‐ and loss‐of‐function assays demonstrated that FGF21 suppressed hypoxia‐induced proliferation of pulmonary artery smooth muscle cells partially through upregulation of H19. In addition, FGF21 deficiency markedly exacerbated hypoxia‐induced increases of pulmonary artery pressure and pulmonary vascular remodelling. In addition, AAV‐mediated H19 overexpression reversed the malignant phenotype of FGF21 knockout mice under hypoxia expose. Further investigation uncovered that H19 also acted as an orchestra conductor that inhibited the function of mechanistic target of rapamycin complex 1 (mTORC1) by disrupting the interaction of mTORC1 with eukaryotic translation initiation factor 4E–binding protein 1 (EIF4EBP1). Our work highlights the important role of H19 in PH treated with FGF21 and suggests a mechanism involving mTORC1/EIF4EBP1 inhibition, which may provide a fundamental for clinical application of FGF21 in PH.

## 1 INTRODUCTION

Pulmonary hypertension (PH) is characterized by progressive vasoconstriction and pulmonary artery remodelling.^1^ Uncontrolled expansion of pulmonary artery smooth muscle cells (PASMCs), pulmonary vascular endothelium damage and excessive extracellular matrix deposition are the common pathological hallmarks of PH.^2^ Despite the availability of treatments that provide symptomatic relief, till date, there has been no effective treatment for PH. Thus, there is still an urgent need to find possible regulatory targets to develop effective anti‐PH agents.

Fibroblast growth factor 21 (FGF21) is a member of the family of endocrine fibroblast growth factors, whose beneficial effects on glucose, lipid metabolism and insulin resistance were found in early studies.^3^, ^4^, ^5^ Recently, mounting studies have reported the protective role of FGF21 in cardiovascular diseases. For example, FGF21 was found to ameliorate diabetes‐induced endothelial dysfunction in mouse aorta^6^ and prevent angiotensin II‐induced hypertension in mice.^7^ However, the role of FGF21 in PH is unclear. Our pre‐experimental research indicated that FGF21 protein expression levels were downregulated in pulmonary arterioles, serum and lung tissues in rats with hypoxia‐induced PH (HPH).^8^ Exogenous administration of FGF21 relieved PH and right ventricular hypertrophy^9^. Therefore, we put forward the following hypothesis: The *FGF21* gene expression is reduced when the body responds to hypoxic stimulation and may act as a protective factor in the pathological process of HPH. In this study, *FGF21* knockout mice were used to further study its function *in vivo*. In addition, the underlying mechanism in PH treated with FGF21 is thought to be complex and has most certainly attracted our great interest.

Recently, numerous studies on non‐coding RNAs have involved them in the pathogenic mechanisms of many diseases.^10^, ^11^, ^12^ Long non‐coding RNAs (LncRNAs) are a class of diverse non‐coding RNA transcripts with a length of more than 200 nucleotides (nt), and no protein coding potential.^13^ The role of lncRNA in PH has attracted widespread interest, which has led to the extensive use of high‐throughput sequencing to characterize the transcriptome profile of lncRNAs associated with PH.^14^, ^15^ Numerous studies have shown that various lncRNAs, such as lncRNA MEG3 and lncRNA MANTIS, play a key role in the occurrence and development of PH.^16^, ^17^ In the present study, changes in lncRNA expression profiles after treatment with FGF21 in PH were detected by RNA‐sequencing. Among the top upregulated lncRNA transcripts after treatment with FGF21, lncRNA H19 (H19) was validated and selected for further study. H19 is a highly conserved imprinted gene located in the 11p15·5 region of chromosome.^18^ Studies by Li et al.^19^ and Maegdefessel et al.^20^ revealed that H19 is implicated in the regulation of pathophysiological processes, such as hypoxia and apoptosis in cardiomyocytes and abdominal aortic smooth muscle cells. Thus, we hypothesized that H19 may play an important role in PH treated with FGF21.

However, little is known about the regulatory role and mechanisms of H19 in PH treated with FGF21. In this study, we found that FGF21 promotes H19 expression, and then, H19 acts as a key protective factor, involved in the treatment of PH by FGF21. We also investigated the effect of H19 in PH treated with FGF21 through functional verification, and identification of downstream mechanisms involved.

## 2 MATERIAL AND METHODS

### 2.1 Animals and HPH models

C57BL/6J background *FGF21* KO mice were obtained as a gift by Dr. Steve Kliewer (University of Texas Southwestern Medical Center, Dallas, TX, USA). Male C57BL/6 mice, and male Sprague–Dawley rats were purchased from the Animal Center of the Chinese Academy of Sciences (Shanghai, China). All animals were allowed to acclimatize to the specific pathogen‐free animal facility for at least one week before use. All procedures were conducted in strict compliance with relevant regulations approved by the Animal Ethics Committee of Wenzhou Medical University.

The construction of HPH mouse model was described previously.^21^ Briefly, C57BL/6 mice were randomly divided into seven groups (*n*  =  6 per group), including N, N+F21 KO, H, H+F21, H+F21 KO, H+AAV‐H19 and H+F21 KO+AAV‐H19. FGF21 was obtained from Prospec (CYT‐281, Prospec, Rehovot, Israel). Briefly, FGF21 (1 mg) was dissolved in 10 ml 0·1% bovine serum albumin (BSA) solution. Then, the medicine was injected intraperitoneally into mice at the prescription dose of 200 μg/kg/day before entering the hypoxia chamber. The AAV virus was constructed and injected into the mouse through the tail vein at a dose of 1 × 10^11^ viral genome (vg)/mouse at the first day, and repeated at the 14th day. All the hypoxia groups were housed in a normobaric hypoxic chamber (9%–11% oxygen concentration) all days for 4 weeks, and all the normoxia groups were exposed to room air. All mice were provided access to food and water freely.

### 2.2 Invasive hemodynamic and RV hypertrophy measurements

Invasive haemodynamics measurements were measured as described.^9^ To assess the mean right ventricular pressure (mRVP) and the mean carotid arterial pressure (mCAP), a Bio Amp, and a PowerLab 8/35 multichannel biological signal recording system (AD Instruments, AUS) were used. To assess the RV hypertrophy, the right ventricle was dissected from the left ventricle and interventricular septum, and then, these dissected samples were weighed to obtain the ratio of right ventricle weight to left ventricle plus interventricular septum weight (RV/LV+S) as well as the ratio of right ventricle weight to body weight (RV/BW).

### 2.3 Tissue staining

10‐μm‐thick paraffin‐embedded lung tissue blocks were sectioned as described previously.^9^ Haematoxylin‐Eosin (HE) staining was performed according to the routine protocols. The pulmonary arteries were selected randomly (external diameters of 25–100 mm) with a Nikon inverted microscope (Nikon, Tokyo, Japan). We calculated the ratios of the pulmonary artery wall area to the total area (WA/TA) and the ratios of the wall thickness to the total thickness (WT/TT) to reflect pulmonary arterial remodelling by Image‐Pro Plus 6·0 (Media Cybernetics, USA). Masson staining was performed according to the manufacturer's instructions (Masson's Trichrome Stain Kit, Solarbio, Beijing, China). The ratio of the collagen area to the total area of the recording area was calculated to reflect the degree of collagen deposition. Six mice were selected and scored for WA/TA, WT/TT and collagen deposition.

### 2.4 Cyto‐immunofluorescence detection

For immunofluorescent staining, rPASMCs seeded on glass coverslips were fixed using 4% paraformaldehyde (PFA) for 30 min at RT. The cells were permeabilized and blocked in 0.1% Triton X‐100, and 5% BSA for 60 min at RT. Subsequently, cells were mildly incubated overnight at 4°C with Ki67 (1:500, AF0198, Affinity) and MYH11 (1:200, ab53219, Abcam) antibodies. Next day, the cells were incubated with a mixture of Alexa Fluor 488 donkey anti‐rabbit (A21206), and Alexa Fluor 546 donkey anti‐mouse (A10036) (all 1:1000, Invitrogen) antibodies in a dark room for 60 min at RT. The cells were then incubated in DAPI (1: 1000, Sigma) for 5 min. Images were acquired using a fluorescence microscope (Olympus, Tokyo, Japan). The ratio of Ki67^+^ cells over total rPASMCs of the recording area was calculated and analysed.

### 2.5 RNA isolation and qRT‐PCR

Total RNA was extracted from the lung tissue, and rPASMCs using Trizol (Invitrogen), and chloroform. Relative cDNA was synthesized from 1 mg of RNA using the iScript cDNA Synthesis Kit (Bio‐Rad, USA). The lncRNAs and gene expression were measured using qRT‐PCR (CFX96 Real‐Time System, Bio‐Rad, USA). β‐actin or GAPDH served as endogenous controls. The relative fold change was calculated using the 2^–△△CT^ method. The primer sequences were shown in Table 1.

**TABLE 1:** Primers used for qRT‐PCR in this study

Gene/Serial number	Primer sequence
NR_130974.1 (H19)	F:5′‐GCTCCACTGACCTTCTAAACGA−3′ R: 5′‐GACGATGTCTCCTTTGCTAACTATC−3′
XR_001781760.1	F: 5′‐CCTTGCGACAGTGGTAGAATCAC−3′ R: 5′‐TATAAGGCAGAGCGTGAGGGAC−3′
ENSMUST00000126427	F: 5′‐ATCCTCCTCCTTCCTAGTCATGG−3′ R: 5′‐GGAGACAGGGCAGGACTTTTAG−3′
ENSMUST00000195990	F: 5′‐GCTGAGGATATAGCCAAGTGGAGA−3′ R: 5′‐TGGAACGGGACTGTTAGCTAGG−3′
ENSMUST00000148335	F: 5′‐CCCACTCTGTGATCTCCTACGA−3′ R: 5′‐TGCGGGACTTTGGTGTCTTC−3′
NR_040442.1	F: 5′‐AGAAAACACAACGACACAGCCC−3′ R: 5′‐AGCTCTACTCGCATTCTTCACGT−3′
ENSMUST00000212043	F: 5′‐GCCACCATTAAGCAGCGACA−3′ R: 5′‐GGTAAGCTGCCGCAGAGTTC−3′
ENSMUST00000190311	F: 5′‐CCTCCTTCTAGACGAGGCTTCTT−3′ R: 5′‐TGTCTGTAAAGGTTGTGAACTCCAC−3′
NR_040630.1	F: 5′‐CAATGTGCAGTTGCTGTGAGAG−3′ R: 5′‐CTTCTGCAAGTTGCCTGTGTTT−3′
ENSMUST00000218666.1	F: 5′‐CCCTCCCGAGGTTCTCCATT−3′ R: 5′‐TATATGCACAGAGACCCGCC−3′
β‐actin	F: 5′‐TCAAGATCATTGCTCCTCCTGAG−3′ R: 5′‐ACATCTGCTGGAAGGTGGACA−3′

### 2.6 Western blot detection

Cells, or isolated lung and liver tissues, were lysed with RIPA buffer (Promega, Madison, WI, USA) on ice. Using a FastPrep‐24 5G Instrument (MP Biomedicals, CA, USA) homogenized lung tissues, and then collected the supernatants. Protein concentrations were measured by a Pierce BCA protein assay kit (Thermo Fisher Scientific Inc., Rockford, IL, USA). Separate samples containing 20–40 µg protein by 5–12% SDS‐PAGE, and transferred to PVDF (0·2, and 0·45 μm, Millipore, Billerica, MA, USA). The membranes were blocked in 5% nonfat dry milk for 1·5 h, and incubated overnight at 4°C on the shaker with primary antibodies. After incubating with secondary antibodies. The proteins were treated with ECL reagent (Pierce, WI, USA) for blot detection. The primary antibodies used were as follows: goat anti‐rabbit IgG H&L (1:10000, ab205718, Abcam), rabbit anti‐FGF21 (1:1000, ab171941, Abcam), rabbit anti‐TSC1 (1:1000, ab270967, Abcam), rabbit anti‐phospho‐S6K (Thr389, 1:1000, #9234, Abcam), rabbit anti‐S6K (1:1000, ab32529, Abcam), rabbit anti‐phospho‐mTOR (Ser2448, 1:1000, ab109268, Abcam), rabbit anti‐mTOR (1:1000, ab2732, Abcam), rabbit anti‐phospho‐EIF4EBP1 (Thr37/46, 1:1000, #2855, CST), rabbit anti‐EIF4EBP1 (1:1000, #9644, CST) and rabbit anti‐β‐ACTIN (1:1000, #4970, CST). Experiments were repeated at least 3 times and β‐ACTIN was used as an internal control. The gel image was analysed by Image Lab 6.0. The ‘Adjusted Total Band Volumes’ were then normalized to the densities of the housekeeping gene, β‐ACTIN, of the same lane and blot to obtain relative expression.

### 2.7 Cell culture and transfection

Cell isolation, cultures and hypoxia exposure methods were as previously described.^21^ On reaching 80%–90% confluence, rPASMCs were passaged and subsequently used for experiments at passages 4–6. RPASMCs were 60%–70% confluent at the time of treatment. The siRNAs and plasmids were synthesized by RiboBio Co. Ltd. (Guangzhou, China). Transfection of siRNAs and plasmids was performed with 70%–80% confluent cells using the Lipofectamine 3000 transfection reagent (Thermo Fisher Scientific Inc.). The efficiency of lncRNA knockdown and overexpression were assessed after 24‐h exposure to hypoxia. Moreover, the most efficient one was chosen by qRT‐PCR. The sequences for siRNA, and plasmid used are shown in Table S1.

### 2.8 Cell proliferation assay

A cell counting kit‐8 (CCK‐8) assay (Dojindo, Kumamoto, Japan) was used as previously described.^19^ Briefly, the rPASMCs were subjected to different treatments with or without siRNA, or plasmid, and then counted, seeded into 96‐well culture plates, at a density of 8–10 × 10^3^ cells/well, and cultured for 24 h under hypoxia. The rPASMCs were subsequently treated with 10 μl of CCK‐8 solution for 2–4 h. The absorbance of the plate was measured using a microplate reader Bio‐Rad Xmark spectrophotometer (Bio‐Rad Laboratories, Hercules, CA, USA), at a dual wavelength of 450/550 nm.

### 2.9 RNA sequencing and bioinformatic analysis

RNA isolation and sequencing were performed by RiboBio Co. Ltd. Briefly, RNA was extracted and purified following routine protocols and verified for quality and integrity. The Epicenter Ribo‐Zero rRNA Removal Kit (Illumina, San Diego, California, USA) was used to remove rRNA from total RNA and fragment it to approximately 200 bp. Subsequently, using the NEBNext^®^ Ultra™ RNA Library Prep Kit for Illumina (New England Biolabs, Ipswich, MA, USA) following the instructions of the manufacturer, the purified RNA was subjected to first‐strand, and second‐strand cDNA synthesis, then adaptor ligation, and low‐cycle PCR were used for enrichment. The purified I library products were assessed using the Tapestation 2200 (Agilent), and Qubit^®^2.0 (Life Technologies/Thermo Fisher Scientific Inc.). Then, all libraries were diluted to 10 pM for cluster generation *in situ* on a HiSeq 3000 paired‐end flow cell. After the removal of reads containing adapter sequences, using ploy‐N at low quality from the raw data, the clean reads were obtained. HISAT2 was used to align the clean reads to the mouse reference genome (mm10) with default parameters. Then, for each gene model, HTSeq was used to convert aligned short reads into read counts.

The R package ‘limma’ was used to identify the differentially expressed lncRNAs. The heatmap was generated using the R package ‘heatmap.2’. Venn diagram was used to identify overlapping and non‐overlapping lncRNAs. Phylogenetic analysis was performed using the BEAST v1.8.0 software (http://www.beast2.org). The H19 subcellular localization was predicted using the lncLocator (http://www.csbio.sjtu.edu.cn/bioinf/lncLocator/) databases. The Gene Set Enrichment Analysis (GSEA) software (version 4.0.1) was applied to determine the significantly enriched gene sets. In our study, the hallmark gene sets were used to calculate the normalized enrichment score (NES). Then, the ‘ggplot2’ package was used to plot the co‐regulated pathways.

### 2.10 Double luciferase reporter gene assay

The 293T cell line (ATCC, Manassas, VA, USA) was used to perform a luciferase reporter assay. The gene encoding firefly luciferase was under the control of the CMV promoter and H19 promoter (RiboBio Co. Ltd.). Transduced cell groups (pGL3‐Basic, pGL3‐Basic‐rH19‐promoter and pGL3‐Basic‐EIF4EBP1 reporter) were cultured in 96‐well plates (2 × 10^4^/well), and then transduced with pcDNA3·1 and pcDNA‐H19 plasmids (RiboBio Co. Ltd.) using Lipofectamine 3000. Luciferase activity was measured using a dual‐luciferase reporter assay System (Promega Corporation, Madison, WI, USA) on a microplate luminometer (Promega Corporation) following the manufacturer's protocols. Experiments for each condition were performed in triplicate.

### 2.11 Construction of H19 overexpression adeno‐associated virus vectors

Recombinant adeno‐associated virus vectors of serotype 9 (rAAV9) that expresses mouse H19 was prepared by Taitool Bioscience Co., Ltd (Shanghai, China). Mouse H19 DNA was amplified by polymerase chain reaction from mouse splenocyte complementary DNA, using the primers 5′‐ACCGGGTGTGGGAGGGGGGTG‐3′, and 5′‐ATGACTGTAACTGTATTTATTGATGGACCCA‐30, and then, DNA segment was inserted into the pAAV‐CMV_bGI‐PA plasmid to construct pAAV‐CMV_bGI‐mH19 PA vector. The AAV vector was prepared according to the previously described protocol for transfection with three plasmids, and no adenovirus, with minor changes to use the active deflation system.^22^ In short, 60% fused human embryonic kidney 293 cells were cultured in a large culture vessel with active air circulation, and the provirus transgenic plasmid, AAV‐1 chimeric helper plasmid (p1RepCap) and adenovirus helper plasmid pAdeno (Avigen Inc) co‐transfection. The crude virus lysate was purified by two rounds of caesium chloride 2‐layer centrifugation. The titre of the original virus relative to the plasmid standard was determined by dot blot hybridization, and then, the stock solution was dissolved in HN buffer (50 mmol/L HEPES, pH 7·4, 0·15 mol/L NaCl) before injection. Before entering the closed hypoxia chamber at the 1st day, we injected the above‐mentioned AAV virus into the mouse through the tail vein at a dose of 1 × 10^11^ viral genome (vg)/mouse, and repeated the injection at the 14th day. Haemodynamic data were obtained on the 28th day.

### 2.12 Statistical analysis

All statistical analyses were performed with GraphPad Prism 6·0 (GraphPad Software, San Diego, CA, USA). All data were expressed as mean  ± standard error mean (SEM). All the results presented were represented from at least three independent experiments. Comparisons between two groups were analysed by unpaired two‐tailed Student's *t*‐test, and multiple comparisons were analysed by ANOVA followed by Bonferroni post hoc test. *P* values of <0.05 were considered statistically significant.

## 3 RESULT

### 3.1 FGF21 relieves HPH in vivo

Given that FGF21 shows beneficial effects in cardiovascular disease,^23^, ^24^, ^25^ we first examined the expression level of FGF21 in HPH mice. HPH mice showed decreased hepatic FGF21 expression and plasma FGF21 levels, and exogenous administration of FGF21 increases hepatic and plasma FGF21 levels (Figure S1A‐B, Supplementary material and method). Compared with heathy controls (HCs), circulating FGF21 levels were notably decreased in subjects with high‐altitude PH (HAPH) (Supporting Information Figure [Link], [Link]C). These data demonstrate that FGF21 is downregulated in HAPH patients and plays a role in PH disease.

**FIGURE 1:**
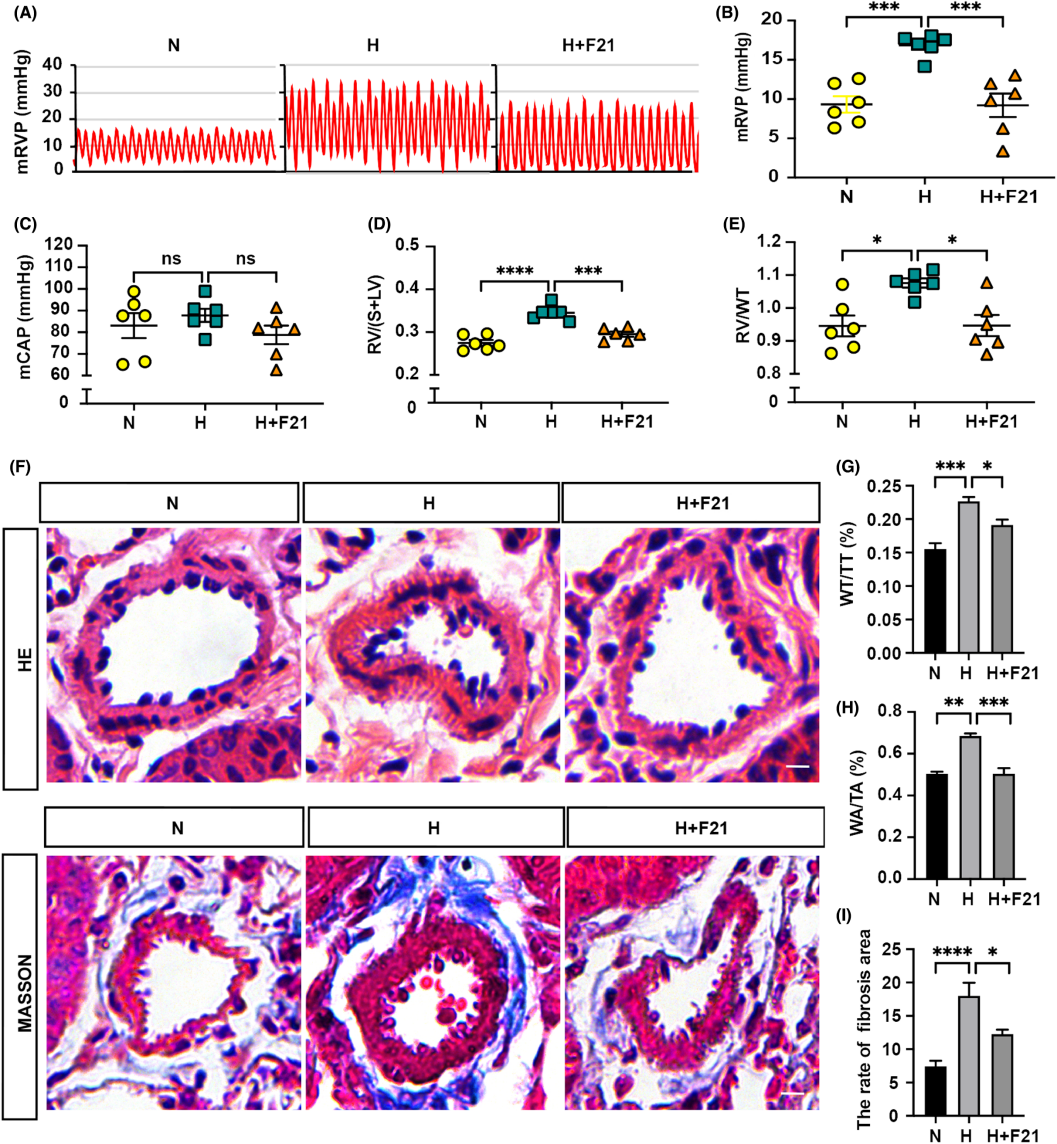
PH amelioration in FGF21 treated mice. (A) Summary of representative pictures of mRVP waves. (B‐C) Quantitative analysis of the mRVP (B), and mCAP (C) in adult C57BL/6 mice of N, H, and H+F21 group (*n* = 6). (D‐E) Quantitative analysis of RV/(S+LV) (D), and RV/WT (E) of each group (*n* = 6). (F) Upper panel: HE staining of pulmonary arteries in lung paraffin sections. Bottom panel: Masson staining of pulmonary arteries in lung paraffin sections. (G‐H) Quantitative analysis of WA/TA (%) (G) and WT/TT (%) (H) of each group (*n* = 6) as shown in (F). (I) Quantitative analysis of the rate of fibrosis area of each group (*n* = 6) as shown in (F). Data are presented as mean ± SEM, ANOVA‐Bonferroni post hoc test, *p < *0·05*, **p < *0·01*, ***p < *0*.*001*, ****p < *0*.*0001*. Scale bars, 20 μm

We next investigate the effect of FGF21 on HPH. The increased mRVP and right ventricular hypertrophy index (reflected by RV/(S+LV) and RV/WT) in hypoxia‐exposed mice compared with normoxia‐exposed mice indicate that the PH model had been successfully established. Administration of FGF21 significantly decreased mRVP in mice, while the mCAP was not affected (Figure 1A‐C). As expected, treatment with FGF21 also significantly reduced hypoxia‐induced right ventricular hypertrophy (Figure 1D‐E). Furthermore, hypoxia‐induced pulmonary artery muscularization, as indicated by the ratios of WA/TA (%), and WT/TT (%), and excessive depositions of collagen around the pulmonary arteries were markedly blocked by replenishment of FGF21 (Figure 1F‐I). Collectively, these results suggest that treatment with FGF21 can largely reverse PH.

### 3.2 Analysis of lncRNA expression profiles in mice treated with FGF21 and identification of H19 in vivo

**FIGURE 2:**
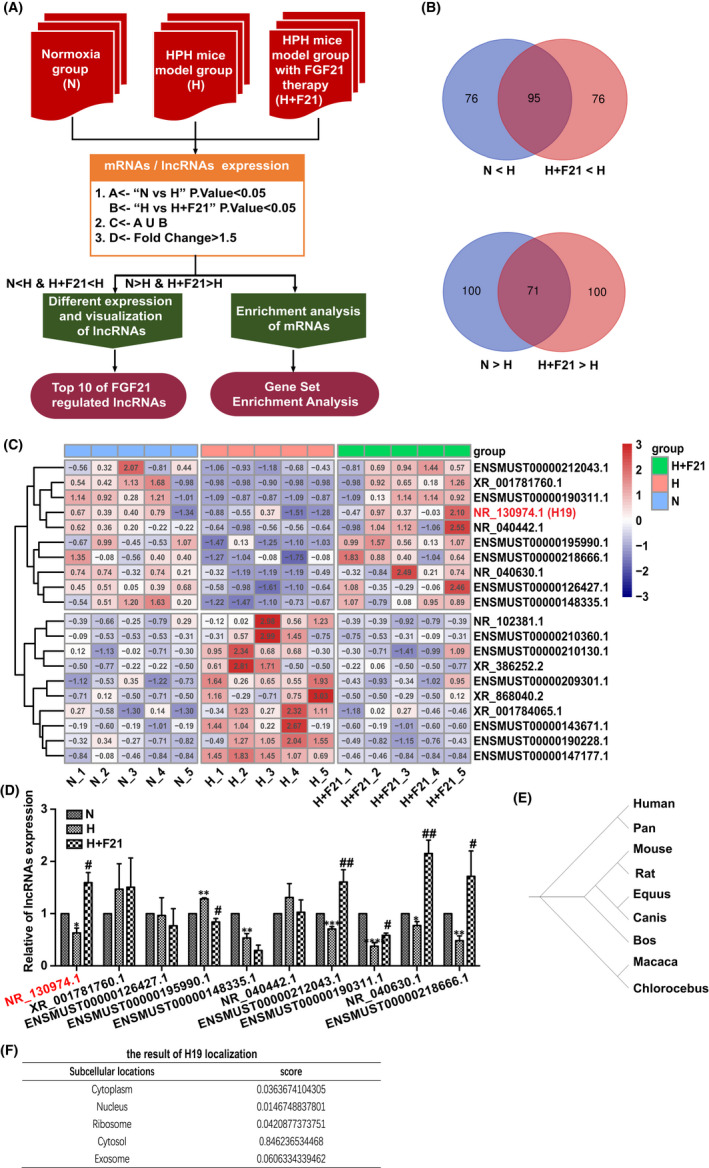
FGF21 promotes H19 transcription under hypoxia exposure *in vivo*. (A) The flow‐process diagram of RNA sequencing data analysis. (B) Venn diagram showed the intersection of differently expressed lncRNAs between N vs H and H vs H+F21. Among the 171 co‐regulated lncRNAs, there were 95 co‐upregulation and 71 co‐downregulation. (C) The heatmap of top 10 co‐upregulated and top 10 co‐downregulated lncRNAs in H group, compared with N group, or H+F21 group respectively. Red represented up‐regulation, and blue represented down‐regulation. The numbers in the heatmap indicated logarithmic expression values in each sample. (D) Validation of RNA sequencing data with qRT‐PCR. The top 10 co‐downregulated lncRNA in H group was shown in bar graph (*n* = 3). (E) Phylogenetic tree of H19, indicated highly sequence conservations of H19. (F) The result of H19 localization in lncLocator. The maximum value was 0·846236534468 indicating that H19 was mainly located in the cytosol. Data are presented as mean ± SEM, ANOVA‐Bonferroni post hoc test, *p < *0·05*, **p < *0·01*, ***p < *0·001*, ****p < *0·0001*

To determine the different lncRNA expression profiles in the normoxia exposure group (N), hypoxia exposure group (H) and hypoxia exposure plus FGF21 treatment group (H+F21), RNA sequencing was performed on lung tissue samples from these mice. The workflow of the bioinformatic analysis is shown in Figure 2A. A total of 171 lncRNAs were screened using the selection criteria (*p* value <0.05, N & H & H+F21), among them 95 were co‐upregulated (N < H & H+F21 < H), 71 were co‐downregulated (N > H & H+F21 > H), and the remaining 5 were excluded (N > H > H+F21, or N < H < H+F21) (Figure 2B). All regulated lncRNAs, and the top 10 co‐regulated lncRNAs screened by two methods (N < H & H+F21 < H, or N > H & H+F21 > H, |Fold Change| >1·5, *p* value <0.05) are shown in the heatmap (Figure 2C, Figure S2 and Table 2). The expression changes of the top10 co‐downregulated lncRNAs (N > H & H+F21 > H) were validated by qRT‐PCR in the tested lung tissue samples (Figure 2D). Among them, H19 (NR_130974.1) was abundantly expressed in the H+F21 group (Table 2). The phylogenetic analysis revealed that H19 is highly conserved in humans, mice and rats (Figure 2E) and is mainly located in the cytoplasm according to lncLocator (Figure 2F). Moreover, H19 has been reported to be a multifunctional lncRNA that regulates cell growth.^20^, ^26^ Thus, we hypothesize that H19 may play an important regulatory role in HPH.

**TABLE 2:** Top10 of FGF21 regulated lncRNA

No.	lncRNA	AveE.N	AveE.H	AveE. F	FC (N/H)	FC (F/H)	P (N/H)	P (H/F)
1.	NR_130974.1(H19)	173.8	89.8	209.4	1.93	2.33	0.05	0.04
2.	XR_001781760.1	81	0.8	73	101.25	91.25	0.01	0.01
3.	ENSMUST00000126427.1	51.4	27	54	1.90	2.00	0.00	0.01
4.	ENSMUST00000195990.1	38.4	24.2	49.2	1.59	2.03	0.01	0.00
5.	ENSMUST00000148335.1	43.4	17	40.8	2.55	2.40	0.01	0.00
6.	NR_040442.1	21.4	11.6	26	1.85	2.24	0.01	0.08
7.	ENSMUST00000212043.1	19.8	10.6	22	1.87	2.08	0.05	0.01
8.	ENSMUST00000190311.1	22.2	1.8	21.4	12.33	11.89	0.02	0.01
9.	NR_040630.1	11.2	3.8	11.4	2.95	3.00	0.00	0.05
10.	ENSMUST00000218666.1	9.6	4.8	10.6	2.00	2.21	0.05	0.03
1.	ENSMUST00000190228.1	28.4	54.6	21.6	0.52	0.40	0.03	0.01
2.	ENSMUST00000210130.1	7.2	16.4	6.8	0.44	0.42	0.05	0.05
3.	XR_001784065.1	7.2	19.4	8.8	0.37	0.45	0.03	0.02
4.	ENSMUST00000209301.1	8.8	25.6	11.8	0.34	0.46	0.01	0.00
5.	NR_102381.1	4.2	13.8	2.6	0.30	0.19	0.03	0.04
6.	ENSMUST00000143671.1	1.4	5	1	0.28	0.20	0.04	0.01
7.	ENSMUST00000210360.1	1.6	7	1.6	0.23	0.23	0.05	0.05
8.	XR_386252.2	1.2	5.8	1.4	0.21	0.24	0.05	0.04
9.	XR_868040.2	1.2	7.2	1.8	0.17	0.25	0.05	0.05
10.	ENSMUST00000147177.1	0.6	5.6	0.4	0.11	0.07	0.00	0.00

### 3.3 FGF21 partially reduces hypoxia‐induced rPASMC proliferation through H19 in vitro

**FIGURE 3:**
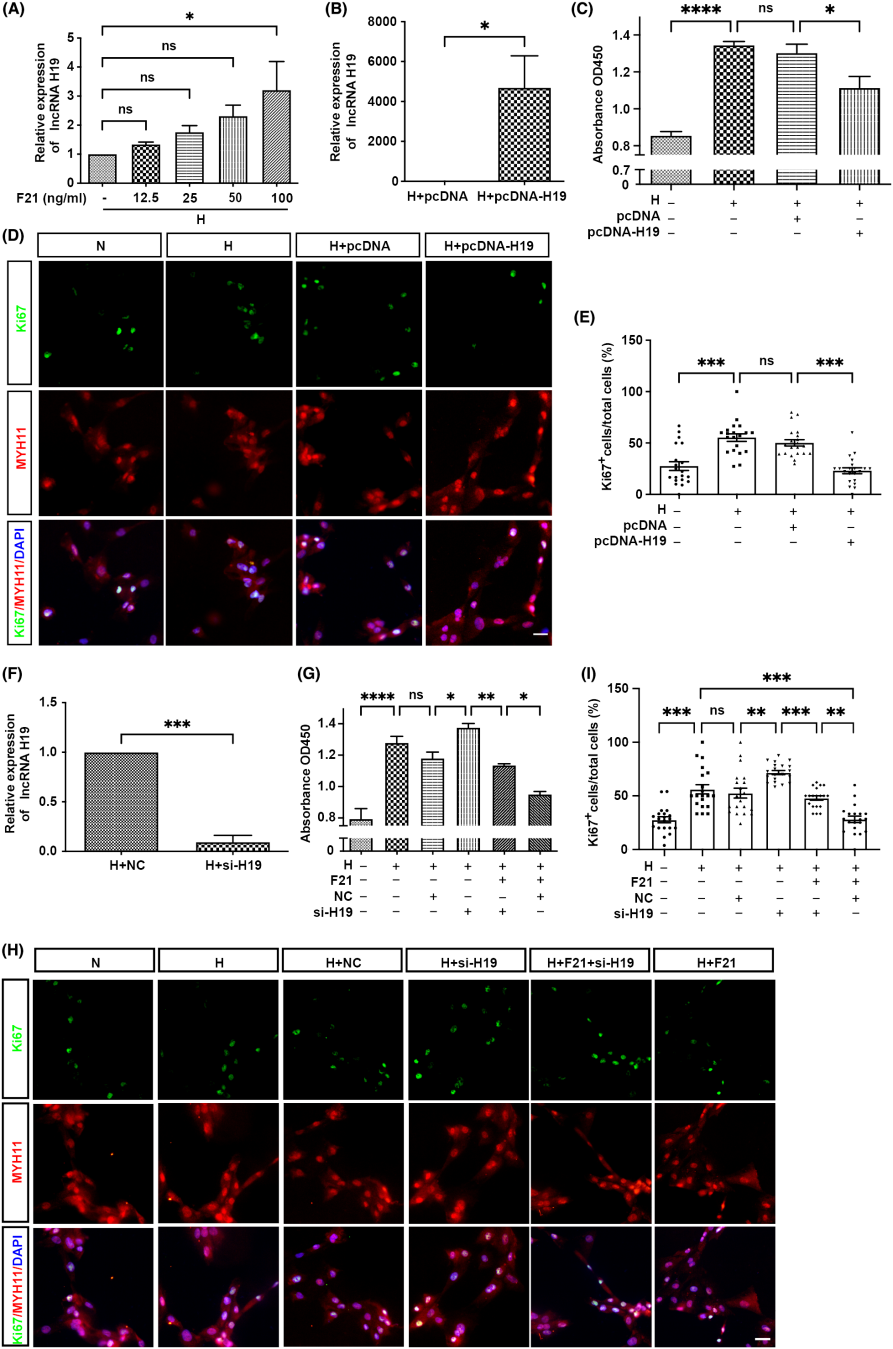
FGF21 partially reduces hypoxia‐induced rPASMC proliferation through H19 *in vitro*. (A) qRT‐PCR analysis of H19 expression in cultured rPASMCs under FGF21 (0, 12.5, 25, 50, 100 ng/ml) treatment after hypoxia exposure for 24 h (*n* = 3, compared with H group). (B) Examination of overexpressed efficiency of H19 plasmid under hypoxia condition by qRT‐PCR (*n* = 3, compared with empty vector control, ANOVA‐Bonferroni post hoc test). (C) CCK‐8 assay detected cell viability in rPASMCs transfected with empty vector pcDNA, or H19 plasmid (1 μg) after hypoxia exposure for 24 h (*n* = 3). (D) Double immunostaining of Ki67 (green) and MYH11 (red) in rPASMCs transfected with controls, and H19 plasmid (H) after hypoxia exposure for 24 h. (E) Quantitative analysis of the percentage of Ki67^+^ MHY11^+^ to MHY11^+^cells as shown in (D) (*n* = 20, data depict cumulative results from 4 independent experiments). (F) Examination of the knockdown efficiency of H19 siRNA under hypoxia condition by qRT‐PCR (*n* = 3, compared with empty vector control, ANOVA‐Bonferroni post‐hoc test). (G) CCK‐8 assay detected cell viability in rPASMCs transfected with siRNA control, or H19 siRNA (20 nmol/L) or FGF21 (100 ng/ml) after hypoxia exposure for 24 h (*n* = 3). (H) Double immunostaining of Ki67 (green), and MYH11 (red) in rPASMCs transfected with controls, or siRNA, or FGF21 after hypoxia exposure for 24 h. (I) Quantitative analysis of the percentage of Ki67^+^ MHY11^+^ to MHY11^+^cells as shown in (H) (*n* = 20, data depict cumulative results from 4 independent experiments). Data are presented as mean ± SEM, ANOVA‐Bonferroni post hoc test, *p < *0·05*, **p < *0·01*, ***p < *0·001*, ****p < *0·0001*. Scale bars, 20 μm

To further study the functional role of H19 in HPH, we additionally examined H19 expression in rPASMCs. The qRT‐PCR analysis results showed that H19 was upregulated by FGF21 in a dose‐dependent manner in the range from 12.5 to 100 ng/ml after exposure to hypoxia for 24 h (Figure 3A), suggesting that H19 may mediate the therapeutic effect of FGF21. Then, gain‐ and loss‐of‐function studies were performed. The efficiency of H19‐knockdown or H19‐overexpression was verified by qRT‐PCR (Figure 3B,F). Cell viability and proliferation rates were higher in hypoxia groups than in controls, but this phenomenon was alleviated by overexpression of H19 (Figure 3C). Moreover, the overexpression of H19 downregulated the hypoxia‐induced expression of Ki67 in MHY11^+^ rPASMCs (MHY11 is a marker of PASMC phenotype) (Figure 3D‐E). In contrast, knock down of H19 significantly increased cell viability, and proliferation rates (Figure 3G), and Ki67 level (Figure 3H‐I). These phenomena were partially reverted by treating cells with FGF21 (Figure 3G‐I). Overall, these results indicate that FGF21 partially reduces hypoxia‐induced rPASMC proliferation through H19 in vitro.

### 3.4 Hypoxia‐induced PH augmentation in FGF21 KO mice

**FIGURE 4:**
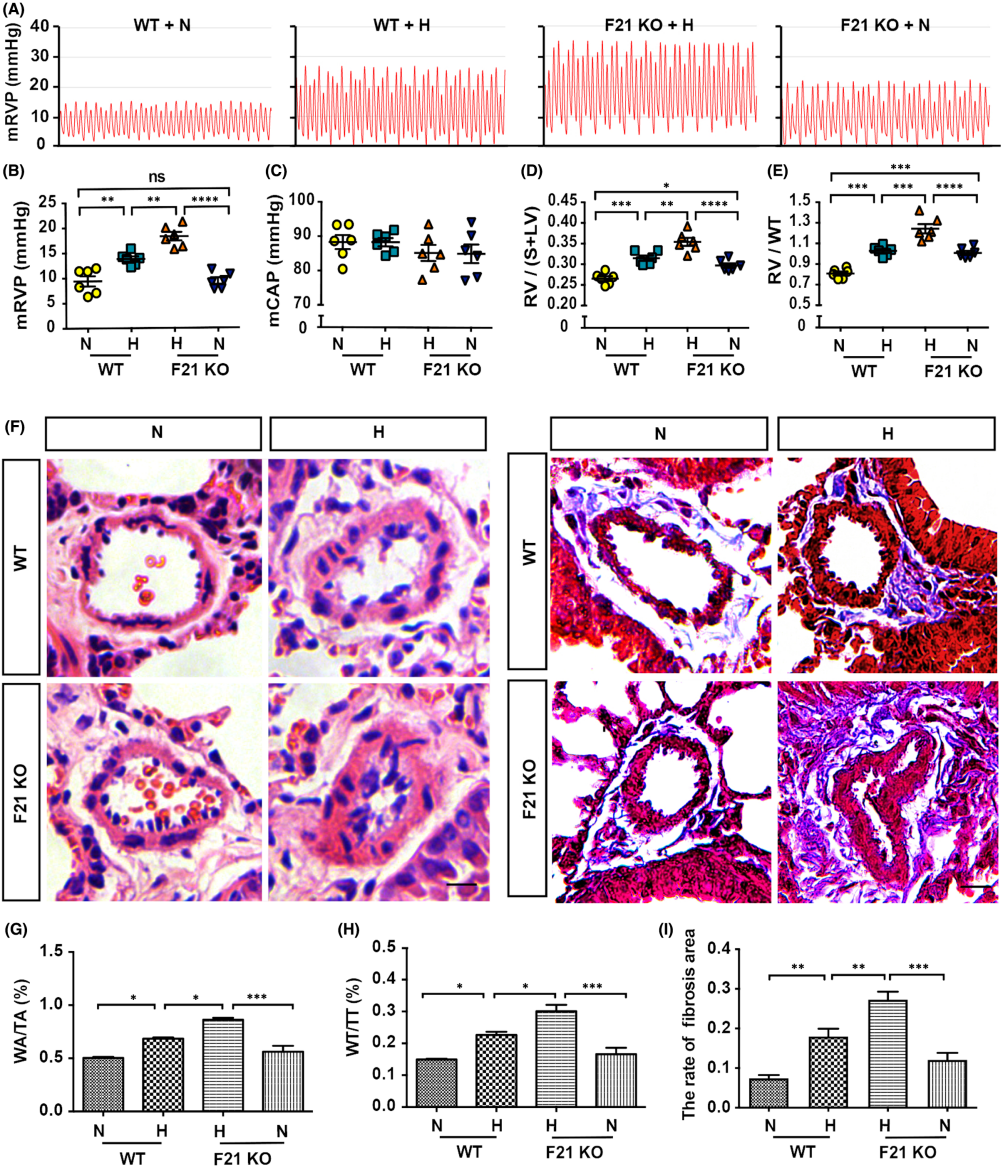
PH augmentation in FGF21 KO mice. (A) Summary of representative pictures of mRVP waves. (B‐C) Quantitative analysis of the mRVP (B) and mCAP (C) in FGF21 knockout adult mice (F21 KO), and their littermates (WT) under normoxia, or hypoxia conditions (*n* = 6). (D‐E) Quantitative analysis of RV/(S+LV) (D), and RV/WT (E) of each group (*n* = 6). (F) Left panel: HE staining of pulmonary arteries in lung paraffin sections. Right panel: Masson staining of pulmonary arteries in lung paraffin sections. (G‐H) Quantitative analysis of WA/TA (%) (G), and WT/TT (%) (H) of each group (*n* = 6) as shown in (F). (I) Quantitative analysis of the rate of fibrosis area of each group (*n* = 6) as shown in (F). Data are presented as mean ± SEM, ANOVA‐Bonferroni post hoc test, *p < *0·05*, **p < *0·01*, ***p < *0*.*001*, ****p < *0*.*0001*. Scale bars, 20 μm

In this study, *Fgf21* KO mice on a C57BL/6 background were used to determine the role of *FGF21* in the pathogenesis of PH (Figure S3). MRVP was further elevated in *Fgf21* KO mice compared with that in littermate controls under hypoxia conditions, whereas this effect was not observed under normoxia conditions (Figure 4A‐B). There was no obvious difference in the mCAP of *Fgf21* KO mice compared with that in the littermate controls, with or without exposure to hypoxia (Figure 4C). However, RV/(S+LV) and RV/WT in *Fgf21* KO mice exposed to hypoxia were found to be markedly higher than those in littermate controls. These effects were particularly pronounced under hypoxia conditions, but some minor changes were also observed under normoxia conditions (Figure 4D‐E). H&E staining was used to evaluate pulmonary arterial remodelling. The ratios of WA/TA, and WT/TT in *Fgf21* KO mice exposed to hypoxia were significantly increased compared with littermate controls (Figure 4F‐H). In addition, the Masson's trichrome staining (Figure 4F, 4I) revealed that the excessive deposition of collagen around the pulmonary arteries is associated with the exposure to hypoxia. Together, these results indicated that the loss of FGF21 markedly exacerbates PH.

### 3.5 H19 overexpression reverses the malignant phenotype of PH in wild‐type (WT) and FGF21 KO mice

**FIGURE 5:**
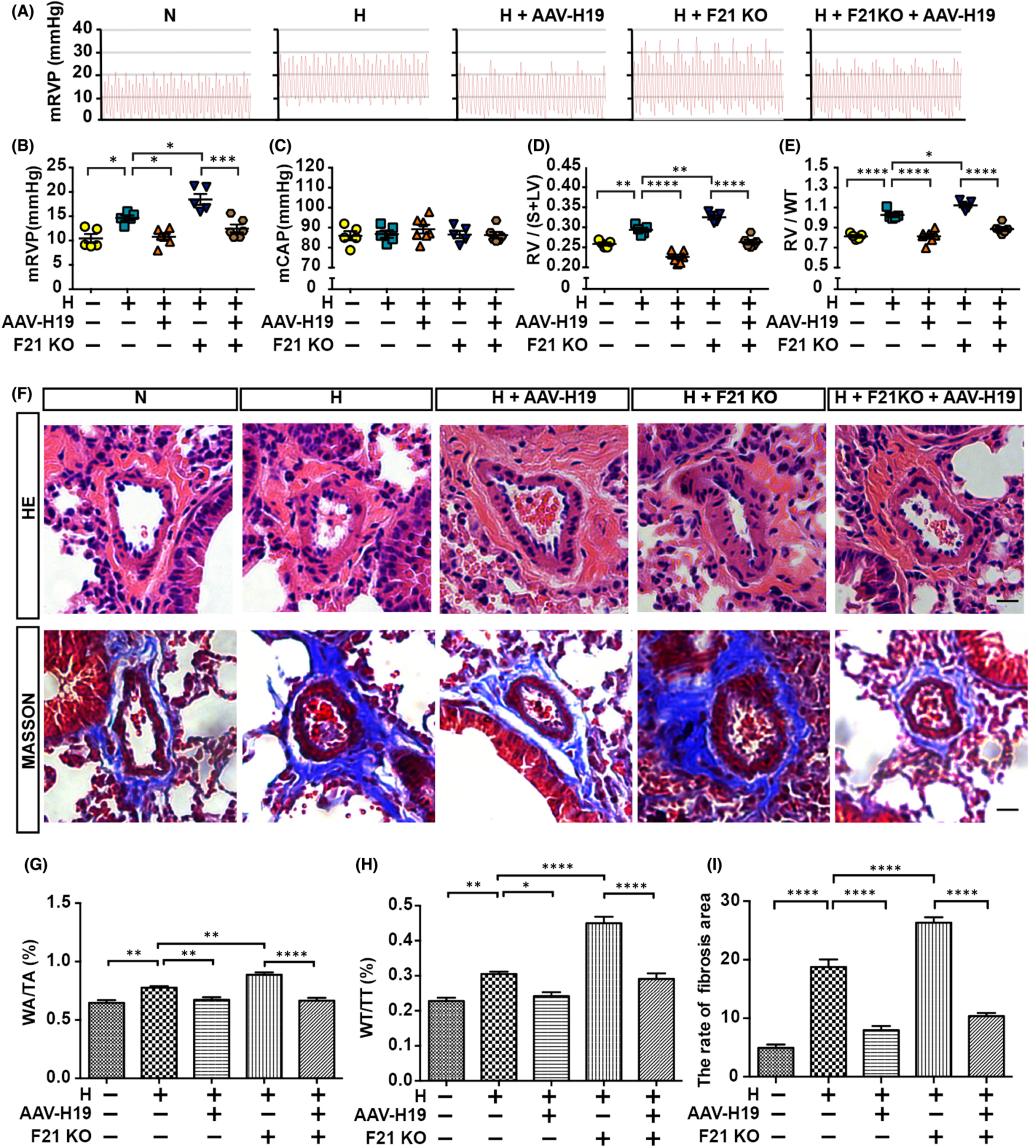
Overexpression of H19 reverses the malignant phenotype of PH in WT and FGF21 KO mice. (A) Summary of representative pictures of mRVP waves. (B‐C) Quantitative analysis of the mRVP (B) and mCAP (C) in adult mice of N, H, H+AAV‐H19, H+F21 KO and H+F21 KO+AAV‐H19 group (*n* = 5–6). (D‐E) Quantitative analysis of RV/(S+LV) (D) and RV/WT (E) of each group (*n* = 5–6). (F) Upper panel: HE staining of pulmonary arteries in lung paraffin sections. Bottom panel: Masson staining of pulmonary arteries in lung paraffin sections. (G‐H) Quantitative analysis of WA/TA (%) (G) and WT/TT (%) (H) of each group (*n* = 6) as shown in upper panel in (F). (I) Quantitative analysis of the rate of fibrosis area of each group (n = 6) as shown in bottom panel in (F). Data are presented as mean ± SEM, ANOVA‐Bonferroni post hoc test, *p < *0·05*, **p < *0·01*, ****p < *0·0001*. Scale bars, 20 μm

We next assessed whether AAV‐mediated H19 (AAV‐H19) overexpression could reverse hypoxia‐induced PH in WT mice and FGF21 KO mice. As expected, the administration of AAV‐H19 by tail intravenous injection significantly reversed hypoxia‐induced pulmonary pressure elevation (reflected by mRVP) in WT mice and FGF21 KO mice, while the mCAP was not affected (Figure 5A‐C). As anticipated, the increases in RV/(S+LV) and RV/WT under hypoxic conditions were markedly reduced by treatment with AAV‐H19 in WT mice and FGF21 KO mice (Figure 5D‐E). Furthermore, AAV‐H19 overexpression also significantly attenuated hypoxia‐induced pulmonary artery muscularization (Figure 5F‐H) and excessive extravascular collagen deposition (Figure 5F, 5I) in WT mice and *Fgf21* KO mice. Therefore, these results suggest that AAV‐H19 overexpression can reverse the malignant phenotype of HPH in WT mice and *Fgf21* KO mice.

### 3.6 FGF21 inhibits the mTORC1/EIF4EBP1 signalling pathway under hypoxia exposure in vivo

**FIGURE 6:**
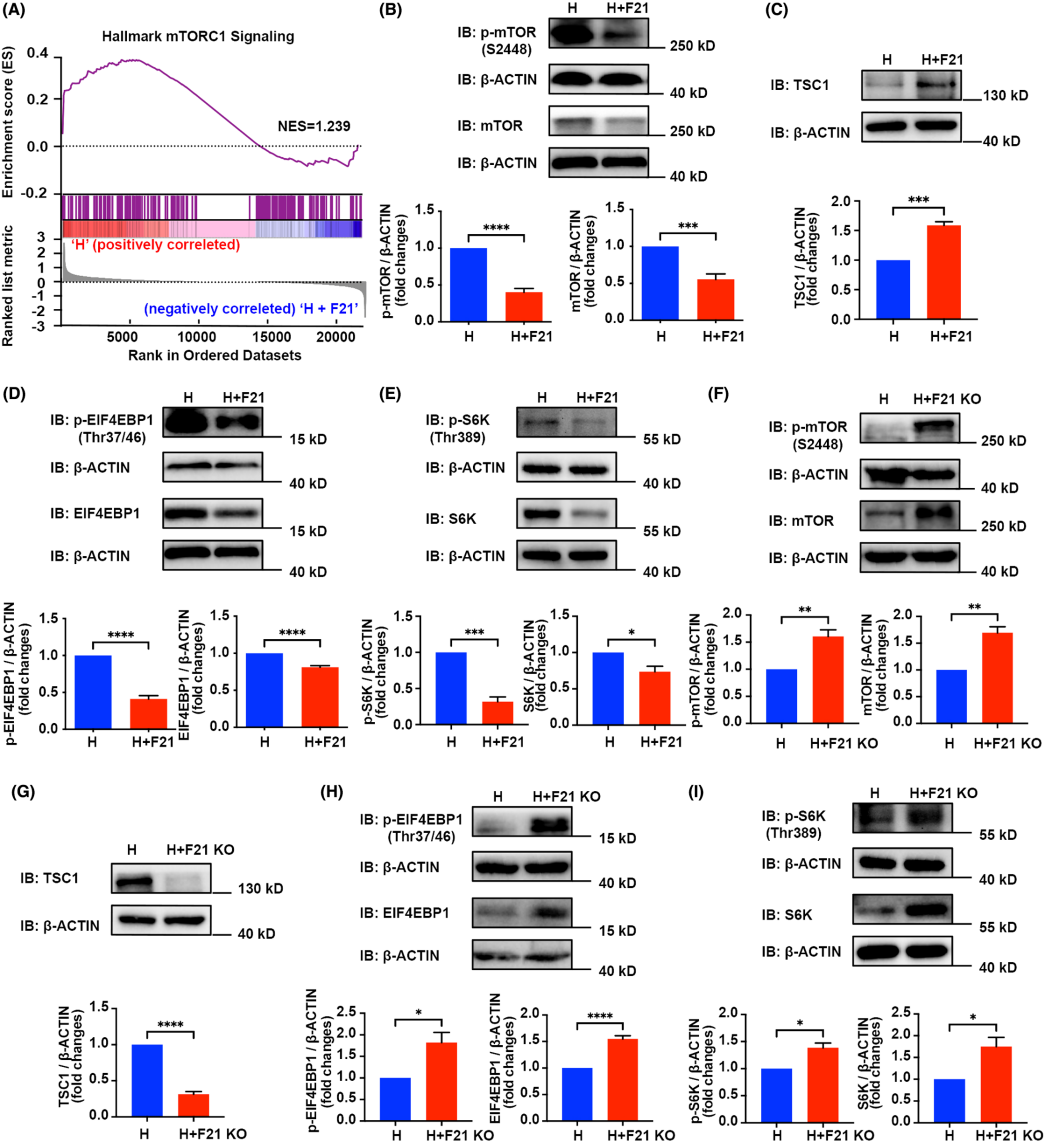
FGF21 inhibit mTORC1/EIF4EBP1 axis *in vivo*. (A) GSEA plot of mTORC1 gene set. The NES was 1·239, indicated mTORC1 signalling pathway was up‐regulated in H group. (B) Upper panel: Western blotting detected p‐mTOR, and mTORC1 expression levels in lung tissue homogenates of H group and H plus F21 group. Bottom panel: Quantitative analysis of p‐mTOR and mTOR expression levels of each group (*n* = 4). (C) Upper panel: Western blotting detected TCS1 expression levels in each group. Bottom panel: Quantitative analysis of TCS1 expression levels of each group (*n* = 3). (D) Upper panel: Western blotting detected p‐EIF4EBP1 and EIF4EBP1 expression levels in each group. Bottom panel: Quantitative analysis of p‐EIF4EBP1 and EIF4EBP1 expression levels of each group (*n* = 4). (E) Upper panel: Western blotting detected p‐S6K, and S6K expression levels in each group. Bottom panel: Quantitative analysis of p‐S6K and S6K expression levels of each group (*n* = 3). (F) Upper panel: Western blotting detected p‐mTOR, and mTORC1 expression levels in lung tissue homogenates of H group, and H plus F21 KO group. Bottom panel: Quantitative analysis of p‐mTOR and mTOR expression levels of each group (*n* = 3). (G) Upper panel: Western blotting detected TCS1 expression levels in each group. Bottom panel: Quantitative analysis of TCS1 expression levels of each group (*n* = 3). (H) Upper panel: Western blotting detected p‐EIF4EBP1 and EIF4EBP1 expression levels in each group. Bottom panel: Quantitative analysis of p‐EIF4EBP1 and EIF4EBP1 expression levels of each group (*n* = 4). (I) Upper panel: Western blotting detected p‐S6K, and S6K expression levels in each group. Bottom panel: Quantitative analysis of p‐S6K, and S6K expression levels of each group (*n* = 3). Data are presented as mean ± SEM, unpaired two‐tailed Student's *t*‐test, compared with H group, *p < *0·05*, **p < *0·01*, ***p < *0·001*, ****p < *0·0001*

The underlying molecular mechanisms responsible for the effects of FGF21/H19 were further investigated by examining the downstream molecular events. We next performed the analysis of hallmark gene sets by GSEA in Hypoxia+FGF21 group compared with the hypoxia‐only counterpart. The mTORC1 pathway was enriched and considered further, since mTORC1 regulates a wide range of cellular processes, including protein synthesis, cell growth, survival, proliferation and metabolism^27^, ^28^, ^29^ (Figure 6A). Consistent with the results of the GSEA, both total protein levels and phosphorylation levels of mTOR, which indirectly reflects the levels of mTORC1, were decreased in HPH mice after treatment with FGF21 (Figure 6B). The tuberous sclerosis complex (TSC1/2) has been established as the major upstream inhibitory regulator of mTOR.^30^, ^31^ We next examined the expression of TSC1 and found FGF21 promoted the protein expression level of TSC1 (Figure 6C). Activated mTORC1 directly phosphorylates one of its main downstream substrates, namely the eukaryotic translation initiation factor 4E binding protein 1 (EIF4EBP1) and the ribosomal S6 kinase (S6K), which play a significant part in the physiological control of translation initiation.^32^ In the HPH mouse model, FGF21 also inhibited the expression of EIF4EBP1, phospho‐EIF4EBP1, S6K and phospho‐S6K (Figure 6D‐E). We further verified the interference of FGF21 on the mTORC1/EIF4EBP1 signalling pathway using *FGF21* KO mice. As anticipated, *FGF21* knockout activated the protein level and phosphorylation level of the mTORC1, EIF4EBP1 and S6K, while decreased the protein expression level of TSC1 (Figure 6F‐I).

### 3.7 FGF21 inhibits the mTORC1/EIF4EBP1 axis via H19 under hypoxia exposure in vitro

**FIGURE 7:**
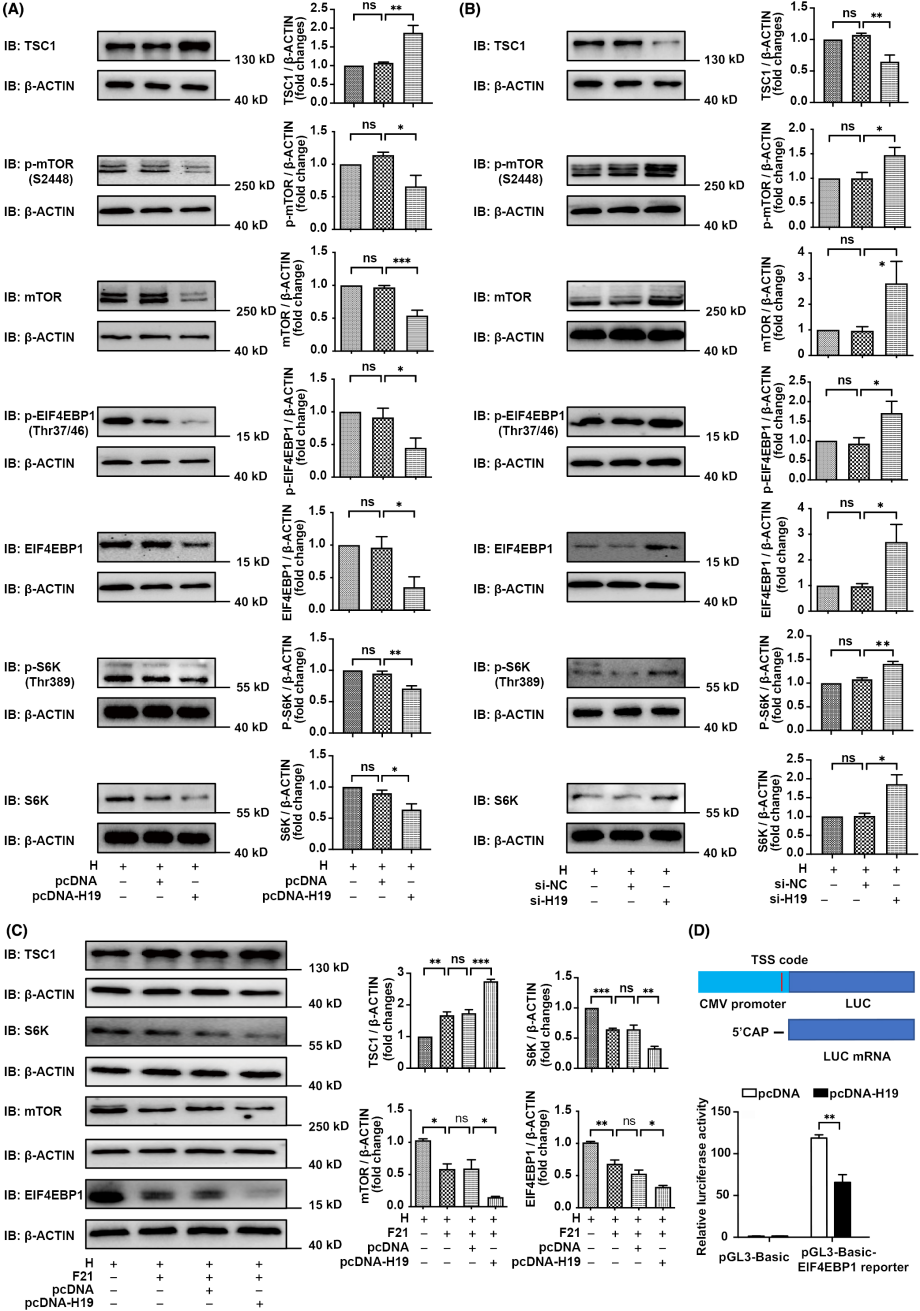
FGF21 inhibit mTORC1/EIF4EBP1 axis via H19 under hypoxia exposure. (A) Left panel: Western blotting detected the expression level of TSC1, p‐mTOR, mTOR, p‐EIF4EBP1, EIF4EBP1, p‐S6K and S6K in rPASMCs transfected with empty vector pcDNA, or H19 plasmid (1 μg) after hypoxia exposure for 24 h. Right panel: Quantitative analysis of TSC1, p‐mTOR, mTOR, p‐EIF4EBP1, EIF4EBP1, p‐S6K and S6K expression levels of left panel (*n* = 3–5, compared with empty vector control, ANOVA‐Bonferroni post‐hoc test). (B) Left panel: Western blotting detected the expression level of TSC1, p‐mTOR, mTOR, p‐EIF4EBP1, EIF4EBP1, p‐S6K and S6K in rPASMCs transfected with siRNA control, or H19 siRNA (20 nmol/L) after hypoxia exposure for 24 h. Right panel: Quantitative analysis of TSC1, p‐mTOR, mTOR, p‐EIF4EBP1, EIF4EBP1, p‐S6K and S6K expression levels of left panel (*n* = 3–6, compared with empty vector control, ANOVA‐Bonferroni post‐hoc test). (C) Left panel: Western blotting detected the expression level of TSC1, mTOR, EIF4EBP1 and S6K in rPASMCs transfected with controls, or H19 plasmid, or FGF21 after hypoxia exposure for 24 h. Right panel: Quantitative analysis of TSC1, mTOR, EIF4EBP1 and S6K expression levels of left panel (*n* = 3–4, compared with empty vector control, ANOVA‐Bonferroni post hoc test). (D) Upper panel: A schematic diagram of the constructions of luciferase reporter gene. Bottom panel: Quantitative analysis of luciferase reporter gene assay. H19‐responsive CMV promoter luciferase reporter was transfected into 293T cells with indicated plasmids (*n* = 4, ANOVA‐Bonferroni post‐hoc test). Data are presented as mean ± SEM, *p < *0·05*, **p < *0·01*, ***p < *0·001*

Through gain‐ and loss‐of‐function assay, we found that phosphorylated and total mTOR, EIF4EBP1 and S6K expression levels were significantly decreased and TSC1 levels were increased by the overexpression of H19 upon exposure to hypoxia (Figure 7A). The knockdown of H19 increased mTOR, EIF4EBP1 and S6K expression, and phosphorylation levels, and decreased the TSC1 expression (Figure 7B). Moreover, the overexpression of H19 enhanced the effect of FGF21 on mTOR, EIF4EBP1 and S6K (Figure 7C).

EIF4EBP1 binds to EIF4E, thus repressing cap‐dependent protein translation at the 5′ cap of the mRNA. In addition, mTORC1 can phosphorylate EIF4EBP1, leading to the separation of EIF4EBP1 from EIF4E, thereby promoting mRNAs translation related to cell survival, proliferation and angiogenesis.^33^ Wu et al. reported that H19 disrupted the interaction of EIF4EBP1, and mTORC1, thus inhibiting mTORC1 activity in pituitary tumours.^33^ To assess whether H19 plays a similar role in HPH, we used a double luciferase reporter gene assay. The firefly luciferase reporter gene was driven by the CMV promoter (CMV‐LUC) to assess the effect of mRNAs translation (Figure 7D). The transcriptional activity of the luciferase reporters containing CMV‐LUC was markedly stronger than that of reporters without CMV‐LUC, thus confirming the efficiency of the LUC system (Figure 7D). In this study, co‐transfection of a plasmid containing H19 significantly reduced the transcriptional activity of CMV‐LUC, suggesting that H19 might interact with the EIF4EBP1 to inhibit mRNA translation (Figure 7D).

Taken together, these results showed that FGF21 inhibits the mTORC1/EIF4EBP1 axis *via* H19 under hypoxic conditions.

## 4 DISCUSSION

Mounting investigations have reported the cardio‐cerebral vascular protective effect of FGF21. Our preliminary data found that exogenous administration of FGF21 effectively relieved HPH in rodent models, which partially fills the gap of FGF21 in PH research.^8^, ^9^ Our research showed that the reduction of FGF21 level in the serum of HAPH patients, as well as in the serum and liver of mice HPH model, which suggesting FGF21 may act as a protective factor. We found that exogenous treatment with FGF21 could reverse hypoxia‐induced pulmonary artery pressure, aggravates pulmonary vascular remodelling and extravascular collagen deposition, whereas FGF21 deficiency exacerbated these negative effects in HPH mice (Figures 1 and 4). These results show that FGF21 is effective against vascular proliferation, inflammatory and hypoxic damage, indicating the potential therapeutic use of FGF21 in PH. Lastly, previous studies have found that FGF21 can restore hypoxia‐induced downregulation of PPARγ,^8^, ^9^ which may be one of the protein regulatory mechanisms through which FGF21 protects against PH. However, the regulatory mechanism of FGF21 is certainly to be quite complicated and much work needs to be performed to understand the underlying molecular mechanisms in PH treated with FGF21.

It is generally believed that lncRNA is related to human gene transcription, translation, modification and expression.^34^ Increasing research data show that lncRNAs regulate several important pathophysiological processes at multiple levels and are closely associated with many major diseases.^35^ Liu et al. examined lncRNA and mRNA expression profiles in PH rats receiving, or not receiving metformin treatment,^36^ and Guo et al. found that the PDGF‐BB‐upregulated expression of lncRNA lnRPT could modulate the proliferation of rPASMCs,^37^ suggesting that lncRNAs can be expected to become a key regulator of a protein intervention in the process of PH. Accordingly, we collected mice lung tissue from N, H and H+FGF21 group and performed RNA sequencing. The obtained RNA sequencing data revealed the changes in lncRNA expression in HPH mice with or without FGF21 treatment, and determined that H19 is involved in PH treated with FGF21 (Figure 2). H19 is a highly conserved and imprinted lncRNA with multiple biological functions in different diseases. However, the regulatory role of H19 in HPH remains unknown, and it could be an interesting target to explore further.

A recent study showed that H19 expression is decreased in failing hearts from mice, and humans, and acts as a promising therapeutic target for the treatment of pathological cardiac remodelling.^38^ These findings of decreased H19 expression are consistent with our finding of a decline of H19 expression in HPH mice. In addition, based on the pathological similarity between cardiac remodelling and pulmonary vascular remodelling, we speculate that H19 is an effective biomarker, and therapeutic target for the treatment of PH. Our study showed that FGF21 promoted H19 expression in a dose‐dependent manner, and knockdown of H19 significantly promoted the proliferation of rPASMCs, while the overexpression of H19 reversed this proliferation promoting effect under hypoxia exposure (Figure 3). Consistent with these, Fei et al. found that the melatonin‐upregulated expression of H19 inhibits the proliferation of PAMSCs in monocrotaline (MCT)‐induced PH,^39^ supporting our finding of antiproliferative effects of H19 in PH. Moreover, Li et al.^19^ and Maegdefessel et al.^20^ also reported antiproliferative effects of H19 on cardiomyocytes, and abdominal aortic smooth muscle cells. However, Bonnet et al. demonstrated that H19 is upregulated in decompensated RV from PAH patients, and the MCT‐induced and pulmonary artery banding rats.^40^ Different blood samples (plasma or serum), different models, tissues and cell samples may have caused some discrepancies. These discrepant results likely reveal the different roles of H19 in physiological and pathological processes. In biological systems, H19 can be activated under certain conditions, but performs distinct functions under other conditions. For example, several human studies have found that H19 is overexpressed in hepatocellular carcinoma.^41^, ^42^, ^43^ In contrast, Zhang^44^ and Schultheiss et al.^45^ showed that H19 is downregulated in hepatocellular carcinoma. This discrepancy may be due to different sample size and methods. In this study, the administration of AAV‐H19 clarifies the positive role of H19 in PH treated with FGF21 *in vivo* (Figure 5).

In addition, pathway enrichment analysis of our RNA sequencing data revealed the involvement of the mTORC1 signalling pathway in mediating the effects of FGF21. MTOR is one of the components of mTORC1, and indirectly reflects the levels of mTORC1. The activation of mTORC1 induces cell proliferation, and growth by promoting protein synthesis, ribosome biogenesis, lipid biogenesis and energy metabolism through its downstream targets EIF4EBP1, p70S6K and its substrate S645.^46^, ^47^, ^48^ In this study, FGF21 promoted the expression of H19, which in turn inhibits the mTORC1, EIF4EBP1 and S6K, suggesting that the anti‐proliferation effect of FGF21, and H19 can be attributed to the inhibition of mTORC1 signalling (Figures 6 and 7). The upregulation of FGF21 leads to a decrease of mTOR signalling pathway activity in a mouse model of neurodegeneration, which supports this notion.^49^ In addition, H19 inhibits the mTOR signalling pathway in hepatic fibrosis,^50^ hepatocellular carcinoma^41^ and cerebral ischemic stroke.^51^ These studies have reported similar inhibitory effects on the mTOR signalling pathway by FGF21 or H19 in different diseases.

mTORC1 can be activated through TSC.^52^, ^53^ Qi Gong et al. found that FGF21 inhibited mTORC1 partly through TSC1.^30^ Consistent with their reports, our study found that both FGF21 and H19 promoted the expression of TSC1 (Figures 6 and 7). More studies are needed to clarify this association. Furthermore, mTORC1 controls the translation initiation mainly by phosphorylating EIF4EBP1. Phosphorylated EIF4EBP1 releases EIF4E to enable cap‐dependent mRNA translation. Wu et al. demonstrated that H19 directly interacts with EIF4EBP1, thereby inhibiting EIF4EBP1 phosphorylation and attenuating mTORC1 activity in pituitary tumours.^33^ Consistent with reported studies, we found, through the luciferase reporter gene experiments, that H19 appears to directly bind EIF4EBP1, thereby attenuating the transcription, and translation effects of EIF4EBP1 (Figure 7).

This study emphasis that FGF21 promotes expression of H19, which in turn to inhibits the mTORC1/EIF4EBP1 signalling pathway under hypoxia exposure (Figure S4). This study suggests that FGF21 and H19 could serve as potential targets for the development of a therapy for PH.

## CONFLICT OF INTERESTS

The authors have declared that no competing interest exists.

## AUTHOR CONTRIBUTION

**Xiuchun Li:** Validation (lead); Visualization (lead); Writing – original draft (lead). **Yaxin Zhang:** Validation (equal); Visualization (equal). **Lihuang Su:** Data curation (equal); Validation (equal). **Luqiong Cai:** Data curation (equal); Validation (equal). **Chi Zhang:** Formal analysis (equal); Visualization (equal). **Jianhao Zhang:** Data curation (equal); Validation (equal). **Junwei Sun:** Data curation (equal); Methodology (equal). **Mengyu Chai:** Visualization (equal). **Mengsi Cai:** Investigation (equal); Visualization (equal). **Qian Wu:** Validation (equal); Visualization (equal).

## AUTHOR CONTRIBUTIONS

X.H., L.W. and X.L. contributed to the design of the study. X.L. and Y.Z. performed most of the experiments in vivo and in vitro. X.L., L.S.^1^, L.C., C.Z.^1^, J.Z., J.S. and M.C. contributed to the interpretation of the data and organize the pictures. M.C, Q.W. and C.Z.^1^ executed the bioinformatics analysis and prepared the tables and figures. X.Y., C.Z.^2^ and J.Z. helped to breed mice and did genotyping. X.L. wrote the manuscript. X.H. and L.W. contributed in modifying the original manuscript and provided funding for the study. All authors approved the final version of the manuscript.

## Supporting information

Supplementary Material

Fig S1

Fig S2

Fig S3

Fig S4

Table S1

## Data Availability

The raw data presented in this study will be made available by the authors without undue reservation.
