# Supplementary material for: FGF21 alleviates pulmonary hypertension by inhibiting mTORC1/EIF4EBP1 pathway via H19
Source: J Cell Mol Med. 2022 Apr 19;26(10):3005–21. doi: 10.1111/jcmm.17318 (PMC9097832; doi:10.1111/jcmm.17318)
Supplement: Supplementary file 1 — Supplementary Material [file JCMM-26-3005-s001.docx]

**Supplementary Material and Methods**

**Human clinical blood samples**

9 patients with high-altitude PH (HAPH) were enrolled between April 2019 and June 2019 at the First Affiliated Hospital of Wenzhou Medical University, and Qinghai Golmujianqiao Hospital. And 21 age-matched healthy controls (HCs) were recruited. Blood samples were obtained from HAPH, or HCs in a quiet state. The blood was drawn into blood collection tubes, gently mixed and allowed to stand for 1 h at room temperature (RT). Then the tubes were centrifuged at 4°C within 2 h to separate the serum. The serum was immediately stored at -80°C until use to avoid degradation. All procedures performed in studies involving human participants were approved by the Ethics Committee of the First Affiliated Hospital of Wenzhou Medical University.

**Biochemical parameters**

Mouse serum FGF21, and human serum FGF21 levels were detected with an FGF21 Quantikine Elisa kit (R&D Systems, Minneapolis, MN, USA), and a Human FGF21 immunoassay kit (ImmunoDiagnostics, lnc.), respectively. Blood glucose was measured using ACCU-CHEK active blood glucose meter.
