# Supplementary material for: FGF21 alleviates pulmonary hypertension by inhibiting mTORC1/EIF4EBP1 pathway via H19
Source: J Cell Mol Med. 2022 Apr 19;26(10):3005–21. doi: 10.1111/jcmm.17318 (PMC9097832; doi:10.1111/jcmm.17318)
Supplement: Supplementary file 2 — Fig S1 [file JCMM-26-3005-s006.pdf]

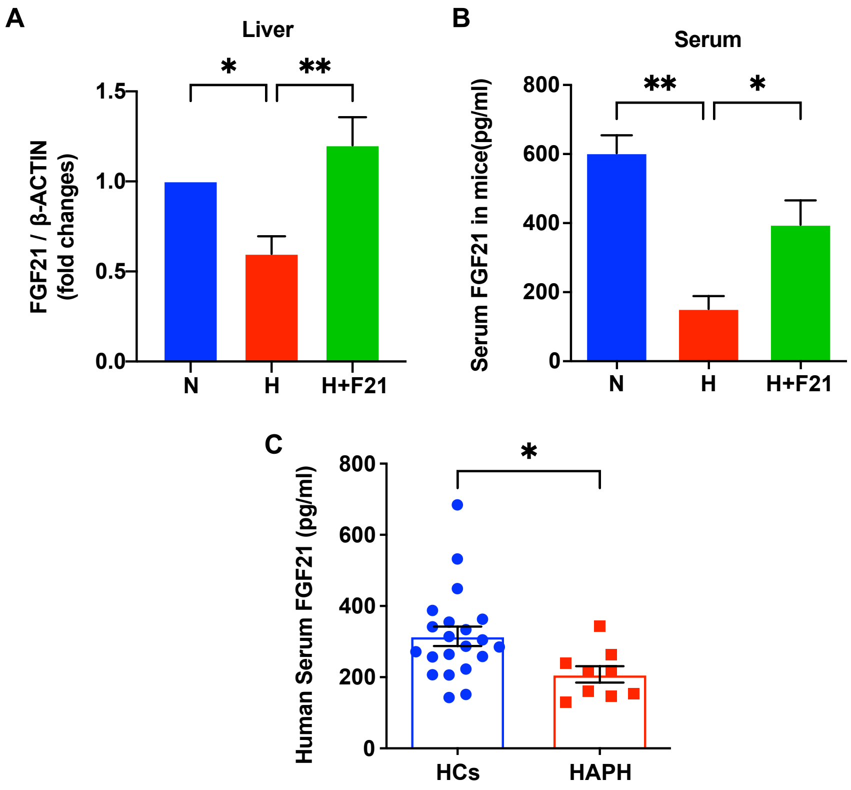

Figure S1 Expression of FGF21 in human serum and experimental PH. (A) WB was used to detect the protein levels of FGF21 in liver tissue homogenates of adult C57BL/6 mice under normoxia, hypoxia, and hypoxia + FGF21. **(B)** ELISA was used to detect the serum FGF21 expression level of adult C57BL/6 mice under normoxia, hypoxia, and hypoxia + FGF21. **(C)** ELISA was used to detect the serum FGF21 expression level of healthy individuals (HCs, n = 21) and HAPH patients (HAPH, n = 9). *$*p < 0.05$, $**p < 0.01$.*
