# Supplementary material for: FGF21 alleviates pulmonary hypertension by inhibiting mTORC1/EIF4EBP1 pathway via H19
Source: J Cell Mol Med. 2022 Apr 19;26(10):3005–21. doi: 10.1111/jcmm.17318 (PMC9097832; doi:10.1111/jcmm.17318)
Supplement: Supplementary file 3 — Fig S2 [file JCMM-26-3005-s003.pdf]

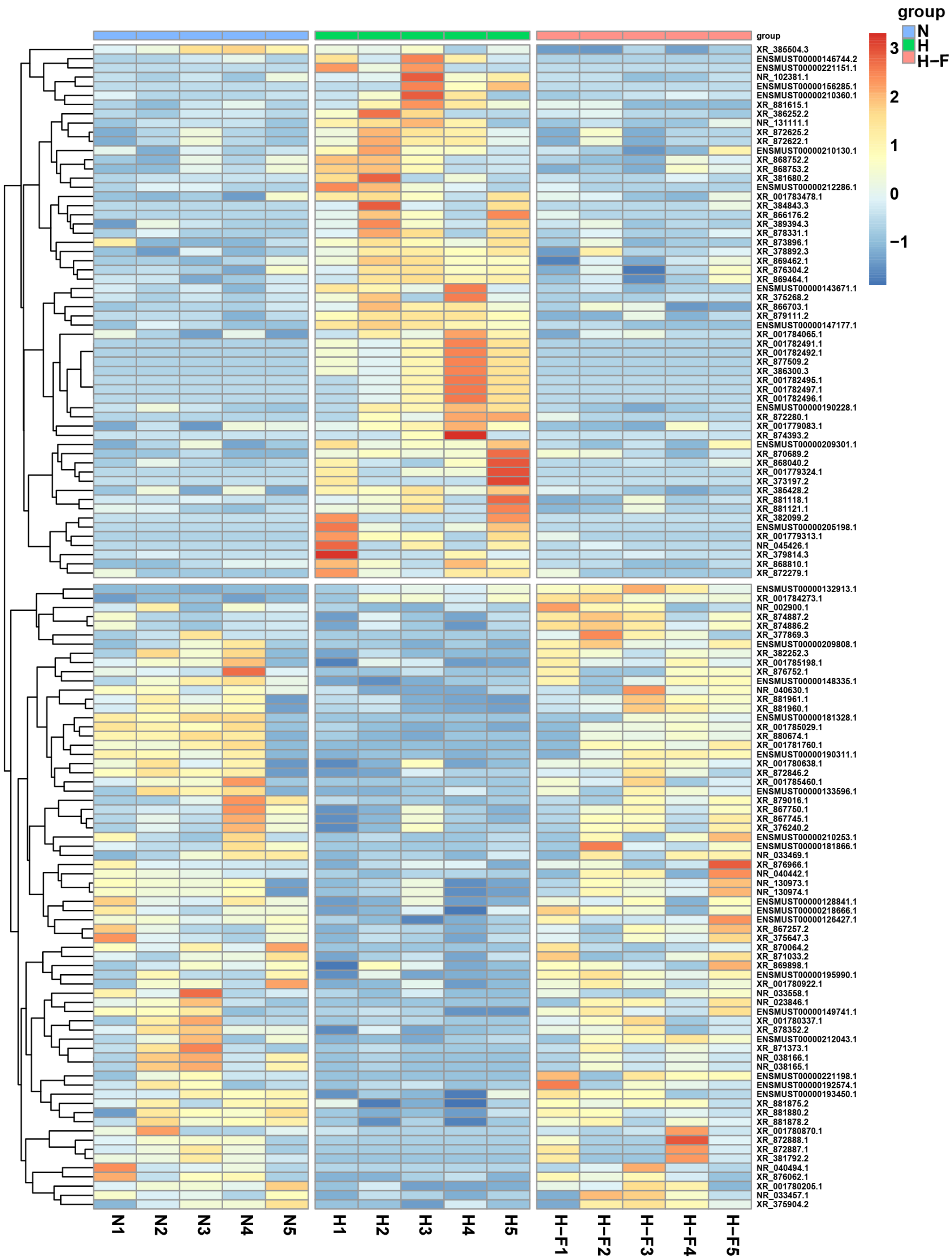

Figure. S2 Identify of differently expressed lncRNAs. The heatmap generated in R using heatmap.2 showed all differentially expressed lncRNAs. Beneath heatmap were the samples enrolled in this study, on the left were the cluster analysis of differentially expressed lncRNAs, on the right of heatmap were differentially expressed lncRNAs. Red represented up-regulation and blue represented down-regulation.
