# Supplementary material for: FGF21 alleviates pulmonary hypertension by inhibiting mTORC1/EIF4EBP1 pathway via H19
Source: J Cell Mol Med. 2022 Apr 19;26(10):3005–21. doi: 10.1111/jcmm.17318 (PMC9097832; doi:10.1111/jcmm.17318)
Supplement: Supplementary file 4 — Fig S3 [file JCMM-26-3005-s002.pdf]

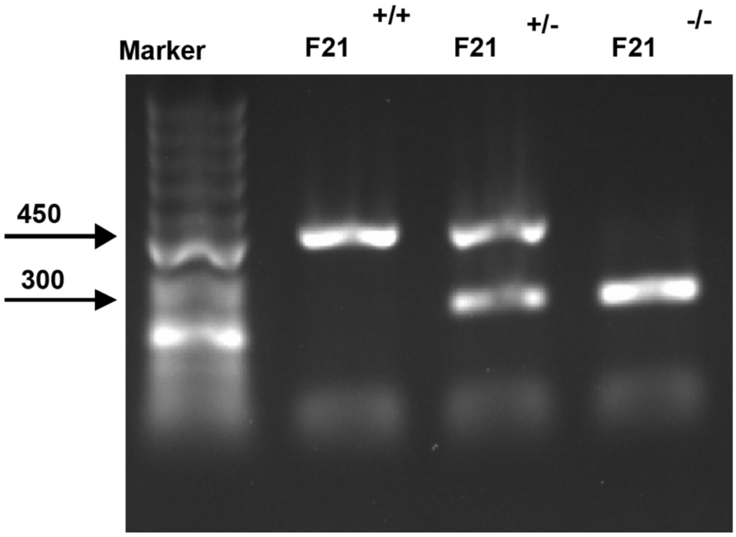

Figure S3 Identify of FGF21 KO mice. Genotype was detected by agarose gel electrophoresis ($FGF21^{+/+}$, $FGF21^{+/-}$, and $FGF21^{-/-}$ from left to right).
