# Supplementary material for: FGF21 alleviates pulmonary hypertension by inhibiting mTORC1/EIF4EBP1 pathway via H19
Source: J Cell Mol Med. 2022 Apr 19;26(10):3005–21. doi: 10.1111/jcmm.17318 (PMC9097832; doi:10.1111/jcmm.17318)
Supplement: Supplementary file 5 — Fig S4 [file JCMM-26-3005-s005.pdf]

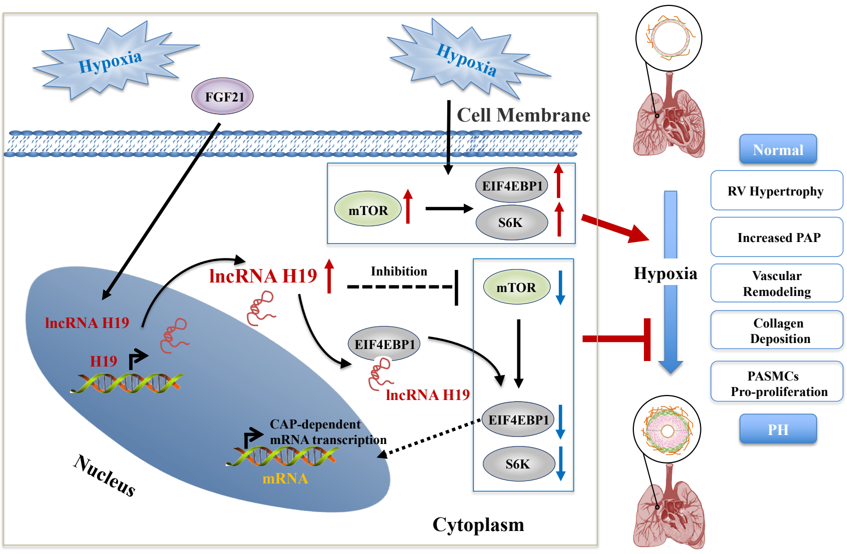

Figure. S4 Diagram of the mechanism of action of FGF21 and H19 against PH. FGF21 promotes the expression of H19, and inhibits the mTOR pathway via activation of H19. The regulatory network of FGF21/H19 eventually reverses increased PAP, RV hypertrophy, vascular remodeling collagen deposition, and PASMCs proliferation, thus attenuates PH.
