# Supplementary material for: FGF21 alleviates pulmonary hypertension by inhibiting mTORC1/EIF4EBP1 pathway via H19
Source: J Cell Mol Med. 2022 Apr 19;26(10):3005–21. doi: 10.1111/jcmm.17318 (PMC9097832; doi:10.1111/jcmm.17318)
Supplement: Supplementary file 6 — Table S1 [file JCMM-26-3005-s004.docx]

**Table S1. Sequences used for siRNA and plasmid transfection and double luciferase reporter gene assay.**

| **Name** | **Sequence** |
| --- | --- |
| si-H19 | 5’-GCAGAATGGCACATAGAAA-3’ |
| pcDNA3.1-H19 | 5’-CCGGGTGTGGGAGGGGGGTGGGGGGTGGGGGTGGGGGGTATCGGGGAAACTGGGGAAGATGGGAGAGCTGGAGGAGAGTCGTGGGGTCCGAGGAGCACCTCGGCATCTGGAGTCTGGCAGGAATGTTGAAGGACTGAGGGGCTAGCTCAGGCAGAGCAAAGGCATCGCAAAGGCTGGAAAACATCGGAGTGAAGCTGAAGGGCCTGAGCTAGGGTTGGAGAGGAATGGGGAGCCAGACATTCATCCCGGTTACTTTTGGTTACAGGACGTGGCGGCTGGTCGGATAAAGGGGAGCTGCTGGGAAGGGTTCGACCCCAGACCTGGGCAGTGAAGGTATAGCTGGCAGCAGTGGGCAGGTGAGGACCGCCGTCTGCTGGGCAGGTGAGTCTCCTTCTTCTCTCTTGGCCTCGCTCCACTGACCTTCTAAACGAAGGTTTAGAGAGGGGGCCTGGTGAGAAGAAGCGGCTGGCCTCGCAGCAGAATGGCACATAGAAAGGCAGGATAGTTAGCAAAGGAGACATCGTCTCGGGGGGAGCCGAGACAGAAGGAGGCTGGGGGACCATTGGCGACCCCAGGTGGAAAGAGCTCTTAGAGAGAAGAAAGAAGAGGTGCAGGGTTGCCAGTAAAGACTGAGGCCGCTGCCTCCAGGGAGGTGATAGGAGTCCTTGGAGACAGTGGCAGAGACCATGGGATCCAGCAAGAACAGAAGCATTCTAGGCTGGGGTCAAACAGGGCAAGATGGGGTCACAAGACACAGATGGGTCCCCAGCCGCCACAACATCCCACCCACCGTAATTCACTTAGAAGAAGGTTCAAGAGTGGCTCTGGCAAAGTCCCAAGTTTGCCAGAGCCTCAATAACTGGAGAATGGAAAAGAAGGGCAGTGCAGGGTGTCACCAGAAGGGGAGTGGGGGCTGCAGGTATCGGACTCCAGAGGGATTTTACAGCAAGGAGGCTGCAGTGGGTCCAGCCTGCAGACACACCATTCCCATGAGGCACTGCGGCCCAGGGACTGGTGCGGAAAGGGCCCACAGTGGACTTGGTACACTGTATGCCCTAACCGCTCAGTCCCTGGGTCTGGCATGACAGACAGAACATTTCCAGGGGAGTCAAGGGCACAGGATGAAGCCAGACGAGGCGAGGCAGGCGGGGCAGAATGAATGAGTTTCTAGGGAGGGAGGTTGGGTGCAGGTAGAGCGAGTAGCTGGGGTGGTGAGCCAGGGAGGCACTGGCCTCCAGAGTCCGTGGCCAAGGAGGGCCTTGCGGGCGGCGACGGAGCAGTGATCGGTGTCTCGAAGAGCTCGGACTGGAGACTAGGGTCTCCAGCAGAGGTGGATGTGCCTGCCAGTCACTGAAGGCGAGGATGACAGGTGTGGTCAATGTGACAGAAAGACATGACATGGTCCGGTGTGATGGAGAGGACAGAAGGGCAGTCATCCAGCCTTCTTGAACACCATGGGCTGGCGCCTTGTCGTAGAAGCCGTCTGTTCTTTCACTTTTCCCAAAGAGCTAACACTTCTCTGCTGCTCTCTGGATCCTCCTCCCCCTACCTTGAACCCTCAAGATGAAAGAAATGGTGCTACCCAGCTCATGTCTGGGCCTTTGAATCCGGGGACTTCTTTAAGTCCGTCTCGTTCTGAATCAAGAAGATGCTGCAATCAGAACCACTACACTACCTGCCTCAGGAATCTGCTCCAAGGTGAAGCTGAAAGAACAGATGGTGTCAACATTTTGAAAGAGCAGACTCATAGCACCCACCCACCCCTGAGAATCCATCTTCATGGCCAACTCTGCCTGACCCGGGAGACCACCACCCACATCATCCTGGAGCCAAGCCTCTACCCCGGGATGACTTCATCATCTCCCTCCTGTCTTTTTCTTCTTCCTCCTTTCCTGTAATTCTGTTTCTTTCCTTTTGTTCCTTCCTTGCTTGAGAGACTCAAAGCACCCGTGACTCTGTTTCCCCATTTACCCCCTTTTGAATTTGCACTAAGTCGATTGCACTGGTTTGGAGTCCCGGAGATAGCTTTGAGTCTCTCCGTATGAATGTATACAGCGAGTGTGTAAACCTCTTTGGCAATGCTGCCCCAGTACCCACCTGTCGTCCATCTCCGTCTGAGGGCAACTGGGTGTGGCCGTGTGCTTGAGGCCTCGCCTTCCCCTCGCCTAGTCTGGAAGCAGTTCCATCATAAAGTGTTCAACATGCCCTACTTCATCCTTTGCCCCTCCTCACCAGGGCCTCACCAGAGGTCCTGGGTCCATCAATAAATACAGTTACAGTCAT-3’ |
| si-GATA1-1 | sense: 5’-GGCACCCAAUGCACUAACUTT-3’  antisense: 5’-AGUUAGUGCAUUGGGUGCCTT-3’ |
| si-GATA1-2 | sense: 5’-GCCUGAGAGUUUGGAUACATT-3’  antisense: 5’-UGUAUCCAAACUCUCAGGCTT-3’ |
| si-GATA1-3 | sense: 5’-CCAAUGCACUAACUGUCAATT-3’  antisense: 5’-UUGACAGUUAGUGCAUUGGTT-3’ |
| pcDNA3.1-GATA1 | 5’-ATGGATTTTCCTGGTCTAGGGGCCCTGGGAACCTCAGAACCCTTGCCCCAGTTTGTGGATTCTGCCCTGGTGTCCTCGACATCAGACTCAGCGGGTTTCTTTTCCTCTGGGCCTGAGAGTTTGGATACAGCATCTTCCTCCACTTCTCCAAATGCAGCCACGGCAGCAGCCACGGCACTGGCCTACTACAGAGAAGCTGAGGCCTACAGACACTCCCCAGTCTTTCAGGTGTACCCGCTGCTCAACAGTATGGAGGGAATCCCAGGGAGCTCACCTTATGCTAGCTGGGCCTATAGCAAGACGGCGCTCTACCCTGCCTCAACTGTATGTCCCAGTCATGAGGATGCCCCTTCCCAGACCCTGGAAGACCCAGATGGGAAGAACAACAATACATTCTTGGAAACCTTGAAGACGGAGCGGCTGAGTCCAGACCTCCTGACCCTGGGGACTGCACTGCCTACATCACTCCCTGTCACCAGCAGTGCTTATGGGGGAGCTGACTTTCCCAGTCCTTTCTTCTCTCCCACTGGGAGCCCTCTCAGCTCAGCAGCCTATTCTTCCCCCAAGTTTCATGGAAGCCTACCATTGGCTCCTTGTGAGGCCAGAGAGTGTGTGAACTGTGGAGCAACGGCTACTCCACTGTGGCGGAGGGACAGGACAGGTCACTACCTGTGCAATGCTTGTGGCTTGTATCACAAGATGAATGGTCAGAACCGGCCTCTCATCCGGCCCAAGAAGCGAATGATTGTCAGCAAGCGAGCAGGCACCCAATGCACTAACTGTCAAACAACAACCACGACCCTCTGGCGGAGGAATGCCAGTGGAGACCCGGTGTGCAATGCCTGCGGCCTCTACTACAAGCTGCACCAGGTGAACCGCCCACTGACCATGAGGAAAGATGGAATCCAGACGAGGAACCGCAAGGCATCTGGAAAAGGGAAAAAGAAGCGGGGCTCAAGTCTGGCCGGAGCAGGAGCGGCTGAAGGACCAGCTGGTGGATTCATGGTGGTAGCTGGTGGCAGCAGTAGTGGGAATTGCGGAGAGGTGGCCCCAGGCTTGACACTGGGCACTGCAGGTACTGCCCATCTCTACCAGGGCCTGGGACCTGTGGTGCTGTCAGGGCCTGTCAGCCATCTTATGTCTTTCCCTGGACCTCTGCTGGGATCGCCTACGGCCTCCTTCCCCACAGGTCCTGTGCCTACCACCACCAGCACTAGCGTAGTGTCTCCACTCAGTTCT-3’ |
| pGL3-Basic-rH19-promoter | 5’-GCTAGTGACAGTGTCGTCTGAAACATCCCGTTCCAGAGACAGCCAAAGTTGGGGTTTGCTGGCGGCAATGTCCAGTCGCCCGCAGCAATGTCCGAAGCCCCTATGCCTCAATATTTGATAAGATTCATAAGGGGTTGGGACACTTGTCTTTCTGGAGGGGTCCCTATGGTCTCTGGATCCCCAAATCAGCCAGTGCGGCTCACTATCAGAAGACGTAGAAGCTGTTGTGTGCACGGGGAAATGGATGTTACCGCGCGGTGGCAGCATACTCCTATATATCGTAGCCCAGATGTAGCCAACTTTTGGGGAGCGATTCACTCCCAGCAATATCCCAAGTCACCCAAACCTGATGCAGTTCATAGGGGTGGTAAGACGTGTGCATCTCTGGAGTGGTTGCACATTGAAACCAGAGAACTTGACTCATTCCCTACACAGCCCGAGATCGTCAATGGCTGGTATCGCCGAAATTGCCGAGCGATGACCAGTTCAATCCCACATACTTTAACACAGAGATGACCAAAGTTGGGGTTCACCTATGGCAAACTCATAGGTCACTCAGGCCTAGCGATTCACAAGGGTCATGGGGTGGTAGGACACACATTTCTCGGGTAACTCCTTCGGTCTTGCGCCCTTCACGATCGGTACACTCCCGACACAGCGCGGATTTGACTATAGCTAGATGGACAAATATGCCGCGTGGTGGCAGTACAACCCTACGTATTGCTGGGCTGACGTGGCATAGATTTGGGGTTCGCCTGTGACAAAGCTTCAAGTCACCCAGGTTCAACAAAGGGGTCAGGCATTTGGATTTATGGAATGGTCCCCTTCTGTCTTGGGACTGCTCGCGCTAGCCAGATCTGCTCAGTCCAAGCGCAATACAAAATCGGTTACGGGTGAGAGACACAGAAATTGCCGCGCATCTCACACGCGGGAACCCCGATAGCCATAAAAAGAGGCTGAGATTCAACTACGGCAATGTCCCAAGTCACCTAGGAACCACAGCGAGAAGTTACAGAGCTGGCATTGGAGACTAGAAGTCAGGGCTCTGCTTAGCTTGAAGCAAAGCGGTGGGGGGGGGGGGTGACGGGGTATAGCGGGTGGGGCATAGGGGTACAGCAGGAGGTAAAGCGGGGGATATAGCGGAGGGTATAGCGGAGGTATAGTGGGAGTATAGCGGGGAGGTATAATGGGGGTATAGCGGGGGGAAGGAGGGATTGCGGGGAGTATAGCAGGGGGTATAGCGGGGGGAAGGGGGTATAGCGGGGAGTATAGCAAGAGGGTATAGCGGGGAGTATGGGGGCTATAGCGGGAGTATAGCAGGGGGTATGGTGGGAGGATGACAGGGGATATAGTGGGGGCTGTAGTGGGGAGGGTATAGTGGGTAATATAGGAGAGTATAGCAGGGTGACTGGGGGGCGGGGGTGACAGGGACCGATTGTCGCCAGACCAGTGAACAGCCCACGTGTGATGGCCAGCCCTCGGTGGTGGTGAGGCTGTCTTTGGGCTGCTGCTGCTGCCAGGGTCCTTAAGGTTCATCAGAAGATGGGGGTCATTCTTTTTCATCTCATATACCGATCTCTTAAGAGCGCAGAGATAGCCACAACCACCACGAGGTGACAGCACAGATTCACACATTACAGCGCCAAGATAACAGAGGCTAGGGTCTACTTACGGCCAAACTGGAGGTGTCTGTCAGTACCATGACTTAGGGTCCACTCACAGCAGTAACCAGGGCTTTTCACAACGTGGGGTTGCTCACAAGAGCCAGAAATGAGCACTCGGCTGACATGCACCTCACCTATGTGAGGACCTGCCGAGTGGTCATGACAGGTCAGCCCTGGGGTCTTCCTCACACCTGAAGCTCCAGTCACTGCCATCCATTCTAACTCTGTGGGTTACAGCCTCCAGGCTTTCAACAGAAGCTAAGGGCAATGGTGTACCACAAGTGGCTAGAGGGACAGGGCATCTTTCCTGTCCCCATTCTATCGCTGATAGTGGTAGGATGGGAGACCATACTGTGCCTCTGGTTGGGTGGGGAGATCAAGGCCAGACTGTGAGGTAACAAATAAAACTCACCGCAGGAGAGTACTGATGAATGTGATGGCCAGAAAAGTGACAGTGAGGAGCACCCAAATTAGCCACCTCGTTGAAACCACTGTGGCCAGACAAGGTCAGTCAAGCAGACTGGGACAAAGGCTGAGGGTTGACCACTGATAAGCTGTTGATACTGCCTGGGACAGTATGAGACCCCTGCCCCCAAATTCCTGTTGGGGTCATTACACAAGCTGAGTAGTAGGCAGCTTCAATGTGGCAGGGTGCCTGGGGCACCACTGTTCCATGAAGGACTCCAGTAGGCTACGGGGCTATATGTTCTCGACCTCAAGGGAGATATTTTGGGACAACGCCAGGCCCTGTCTAAGGGATTCCAAAGTGGGAGTTGTGGTGAGGCTGTCTTTGGAGAATTTCAGGACGAGTGCGGGTGCGGGGTGAGGCGAACGTGCGCTGGAAAGATCCAGGGTGGAGGTGGGCCCTGCGGGGGCCCTGGCGGGTCCTTGTACTGATTGGTTGACAGAGTAGGGGCGGGAATTCTGGGCGGAGCCACTCCAGTTAGAAAAAGCCCGGGCTAGAGGGCCCGAAGCACCGGGTGTGGGAGGGGGGTGGGGGGTGGGGGTGGGGG -3’ |
| pGL3-Basic-EIF4EBP1 reporter | 5’-GACATTGATTATTGACTAGTTATTAATAGTAATCAATTACGGGGTCATTAGTTCATAGCCCATATATGGAGTTCCGCGTTACATAACTTACGGTAAATGGCCCGCCTGGCTGACCGCCCAACGACCCCCGCCCATTGACGTCAATAATGACGTATGTTCCCATAGTAACGCCAATAGGGACTTTCCATTGACGTCAATGGGTGGAGTATTTACGGTAAACTGCCCACTTGGCAGTACATCAAGTGTATCATATGCCAAGTACGCCCCCTATTGACGTCAATGACGGTAAATGGCCCGCCTGGCATTATGCCCAGTACATGACCTTATGGGACTTTCCTACTTGGCAGTACATCTACGTATTAGTCATCGCTATTACCATGGTGATGCGGTTTTGGCAGTACATCAATGGGCGTGGATAGCGGTTTGACTCACGGGGATTTCCAAGTCTCCACCCCATTGACGTCAATGGGAGTTTGTTTTGGCACCAAAATCAACGGGACTTTCCAAAATGTCGTAACAACTCCGCCCCATTGACGCAAATGGGCGGTAGGCGTGTACGGTGGGAGGTCTATATAAGCAGAGCTCTCTGGCTAACTGGAGCCGG-3’ |
